# Cytoplasmic cleavage of *IMPA1* 3′ UTR is necessary for maintaining axon integrity

**DOI:** 10.1016/j.celrep.2021.108778

**Published:** 2021-02-23

**Authors:** Catia Andreassi, Raphaëlle Luisier, Hamish Crerar, Marousa Darsinou, Sasja Blokzijl-Franke, Tchern Lenn, Nicholas M. Luscombe, Giovanni Cuda, Marco Gaspari, Adolfo Saiardi, Antonella Riccio

**Affiliations:** 1 MRC Laboratory for Molecular Cell Biology, University College London, London WC1E 6BT, UK; 2 Francis Crick Institute, London NW1 1AT, UK; 3 UCL Genetics Institute, University College London, London WC1E 6BT, UK; 4 Research Centre for Advanced Biochemistry and Molecular Biology, Department of Experimental and Clinical Medicine, Magna Graecia University of Catanzaro, Catanzaro 88100, Italy

**Keywords:** sympathetic neurons, axons, neuronal development, NGF, local translation, mRNA localization, 3’UTR, 3’UTR cleavage, RNA processing, alternative polyadenylation

## Abstract

The 3′ untranslated regions (3′ UTRs) of messenger RNAs (mRNAs) are non-coding sequences involved in many aspects of mRNA metabolism, including intracellular localization and translation. Incorrect processing and delivery of mRNA cause severe developmental defects and have been implicated in many neurological disorders. Here, we use deep sequencing to show that in sympathetic neuron axons, the 3′ UTRs of many transcripts undergo cleavage, generating isoforms that express the coding sequence with a short 3′ UTR and stable 3′ UTR-derived fragments of unknown function. Cleavage of the long 3′ UTR of *Inositol Monophosphatase 1* (*IMPA1*) mediated by a protein complex containing the endonuclease argonaute 2 (Ago2) generates a translatable isoform that is necessary for maintaining the integrity of sympathetic neuron axons. Thus, our study provides a mechanism of mRNA metabolism that simultaneously regulates local protein synthesis and generates an additional class of 3′ UTR-derived RNAs.

## Highlights

• Axons and cell bodies of sympathetic neurons express distinct 3′ UTR isoforms
• Axon-specific short 3′ UTR isoforms are generated by local cleavage of longer 3′ UTRs
• A protein complex containing Ago2, Upf1, HuD, and Pabpc4 mediates the 3′ UTR cleavage

## Graphical Abstract

**Figure:**
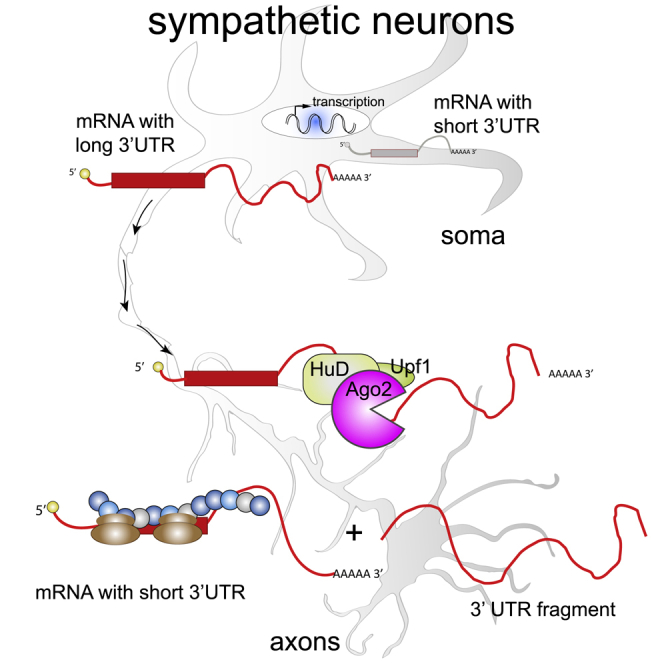

## Teaser

Andreassi et al. show widespread differential usage of 3′ UTR in axons and cell bodies of sympathetic neurons. In axons, the cleavage of a longer 3′ UTR of *Impa1* generates a shorter isoform that is stable, polyadenylated, and necessary for maintaining axon integrity.

## Introduction

Asymmetric localization of RNA is an evolutionarily conserved mechanism that allows spatial restriction of protein synthesis to cellular compartments. In neurons, transcripts are transported to dendrites and axons where they are rapidly translated in response to extracellular cues, such as neurotrophins (Dalla Costa et al., 2020; Holt et al., 2019). In sympathetic and sensory neurons, for example, hundreds of transcripts are targeted to axons in response to nerve growth factor (NGF) (Andreassi et al., 2010; Willis et al., 2005, 2007) in a process that is necessary for both neuronal survival and differentiation and for nerve regeneration after injury (Ben-Yaakov et al., 2012; Terenzio et al., 2018). Critically, incorrect processing and delivery of messenger RNA (mRNA) have been implicated in many neurological disorders to the extent that it has been proposed that all neuronal disorders are fundamentally RNA disorders (Baleriola et al., 2014; Wang et al., 2007).

Eukaryotic mRNAs share common features that include exons and introns; 5′ and 3′ untranslated regions (UTRs); a modified base at the 5′ end named the “cap”; and a stretch of adenosines at the 3′ end named the poly(A) tail, which confers stability to the transcript and prevents premature degradation and enables translation. Information necessary for RNA processing can be stored anywhere along the transcript; however, the elements that regulate mRNA localization and translation are primarily found within the 3′ and 5′ UTRs (Andreassi et al., 2018; Leppek et al., 2018; Mayr, 2017). Much effort has been put into the identification of 3′ UTR sequences that regulate mRNA transport in dendrites and axons. The first localization element was identified within the 3′ UTR of β-actin and named zipcode because it was necessary for delivering β-actin mRNA to the leading lamellae of fibroblasts and to dendrites (Eom et al., 2003; Kislauskis et al., 1994). Following this seminal discovery, many localization elements were found within the 3′ UTRs of transcripts transported to both dendrites and axons. In sympathetic neurons, a 120 nt element within the 3′ UTR is necessary and sufficient to target the *Inositol Monophosphatase 1* (*IMPA1*) transcript to axons in response to NGF (Andreassi et al., 2010). Similarly, a 3′ UTR variant of *importin-β1* is responsible for axonal targeting of the transcript and for activating the injury response in adult sensory neurons (Ben-Yaakov et al., 2012; Perry et al., 2012). In hippocampal neurons, isoforms of the *BDNF* transcript with either short or long 3′ UTRs are localized to dendrites in response to extracellular cues (Allen et al., 2013; An et al., 2008; Will et al., 2013). Despite all of this evidence, how mRNA metabolism is regulated in neurons and the role of the 3′ UTR in determining the translation of transcripts necessary for neuronal functions remain largely unknown.

Here, we show that the 3′ UTR of hundreds of transcripts localized in sympathetic neuron axons are potentially cleaved to simultaneously generate an mRNA isoform carrying the coding sequence and a stable 3′ UTR-derived RNA fragment. Analysis of *IMPA1*, a highly abundant axonal transcript ( Andreassi et al., 2010), revealed that the 3′ UTR is remodeled in axons to generate an isoform that is very efficiently translated in response to NGF and a non-coding RNA that may contribute to the maintenance of axon growth and integrity. Importantly, we discovered that 3′ UTR remodeling is performed by a cleavage complex containing the endonuclease Argonaute 2 (Ago2) and the RNA-binding protein (RBP) ELAV-like protein 4 (HuD).

A handful of recent studies have demonstrated distinct expression patterns of 3′ UTRs and respective coding sequences for thousands of neuronal (Kocabas et al., 2015) and non-neuronal (Mercer et al., 2011) genes. Because these 3′ UTR fragments do not originate from independent transcriptional events (Mercer et al., 2011), it has been suggested that remodeling of 3′ UTRs may occur post-transcriptionally. We provide evidence that 3′ UTR cleavage takes place in sympathetic neuron axons and demonstrate the impact on local protein synthesis and maintenance of axon integrity.

## Results

### Identification of 3′ UTR isoforms in axons and cell bodies of sympathetic neurons

**Figure 1:**
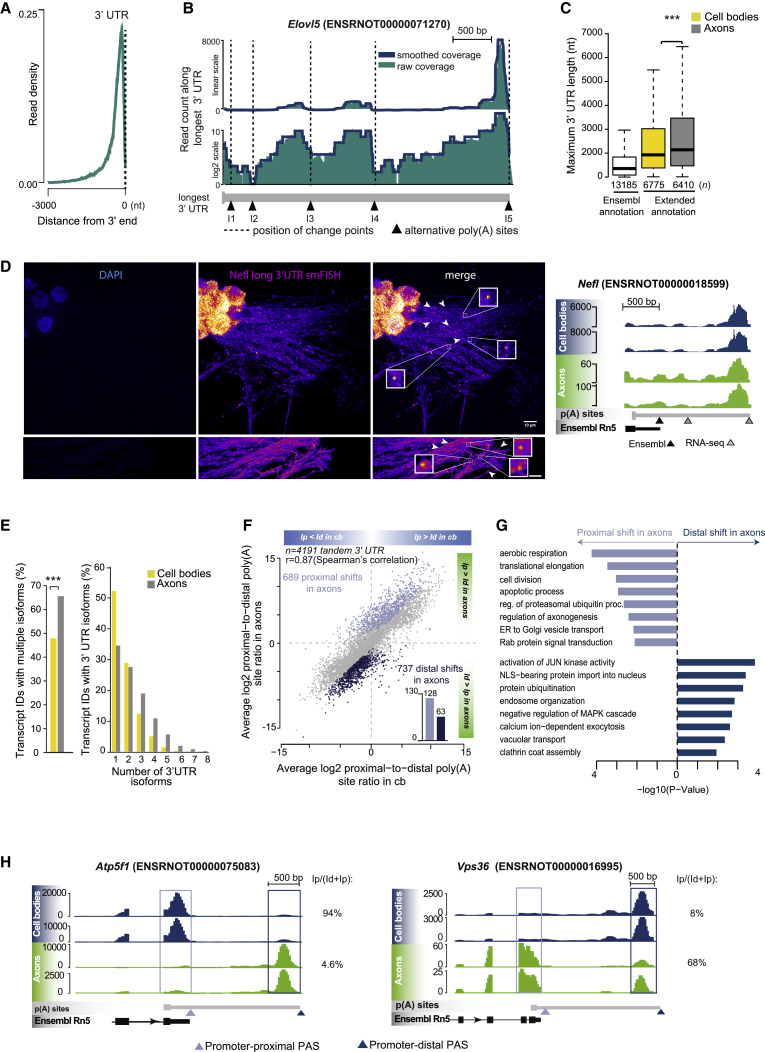
3′ End RNA-seq on RNA isolated from axons and cell bodies (A) Accumulation of reads at the 3′ end of the transcripts. The read density (number of reads/nt divided by the total number of reads) of 4,975 transcripts between 2,000 and 3,000 nt long is shown. Dashed line indicates the 3′ end. (B) Identification of novel 3′ ends in the longest 3′ UTR of *Elovl5*. Raw coverage was smoothed using a running median (window width of 100 nt), and potential 3′ ends were identified by segmenting sudden transitions in read depth. (C) Maximum 3′ UTR lengths for existing annotations in Ensembl Rn5 and for those newly identified by 3′ end RNA sequencing (RNA-seq) in this study (^∗∗∗^p = 1.010881e−06; two-sided Wilcoxon rank-sum test). (D) Left: single-molecule FISH (smFISH) of *Nefl* long 3′ UTR in sympathetic neurons cell bodies and axons. Arrowheads indicate mRNA puncta without any pixel dilation. Insets show 5× magnification of boxed area. Scale bar, 10 μm. Right: genome browser view of *Nefl* in axons and cell bodies. (E) Percentage of cell body and axonal transcript IDs showing multiple 3′ UTRs (left) and distribution of 3′ UTR isoforms per expressed Ensembl transcript ID (right) (^∗∗∗^p = 3.116589e−72; two-sided Fisher’s exact count test). (F) Scatterplot of the relative usage of promoter-proximal and promoter-distal poly(A) sites in cell bodies and axons (FDR < 0.01 between cell body and axonal compartment; Fisher’s exact test). Distal shifts in axons compared with cell body (dark blue); proximal shifts in axons compared with cell body (light blue). (Inset) 3′ UTR isoforms with proximal or distal shift uniquely detected in axons (see STAR methods for details). (G) Statistically enriched GO terms for transcripts showing a proximal (top) or distal (bottom) shift in poly(A) site usage in axons. (H) Genome browser view of representative transcripts with a marked shift toward decreased (*Atp5f1*) or increased (*Vps36*) promoter-proximal poly(A) site usage in axons compared with cell bodies. See also Figure S1.

To characterize the 3′ UTR isoforms localized in cell bodies or axons of rat sympathetic neurons, we performed stranded 3′ end RNA sequencing (RNA-seq) using mRNA isolated from neurons grown in compartmentalized chambers (Figure S1A). In this model system, distal axons are separated from cell bodies by a 1 mm wide Teflon divider, allowing the isolation of mRNA from distinct cellular compartments (Campenot, 1977; Riccio et al., 1997). Prior to sequencing, mRNA was subject to two rounds of linear amplification (Figure S1B) that led to the accumulation of reads at the 3′ end of transcripts independently of the 3′ UTR length (Figures 1A and S1C), generating a read coverage profile similar to poly(A)-seq (Shepard et al., 2011). The rat genome is poorly annotated compared with mouse and human; therefore, 3′ end RNA-seq data were used to identify unknown isoforms by re-annotating the 3′ ends to the Ensembl Rn5 database (v.78). A segmentation algorithm (see STAR methods for details) was used to recognize regions of continuous coverage, which are expected to coincide with genuine 3′ UTRs (Figure 1B). In addition to the existing Ensembl annotations, we identified 26,468 new 3′ UTR isoforms and extended the 3′ UTR of 7,506 transcripts (Figures 1C, S1D, and S1E). We confirmed the expression of isoforms carrying longer 3′ UTRs in axons and cells bodies by using single-molecule fluorescence *in situ* hybridization (smFISH) targeting the long 3′ UTR of three transcripts: *Nefl*, *Snrk*, and *Apba2* (Figures 1D and S1F). The reliability of our annotations was further confirmed by checking them against a comprehensive polyadenylation atlas compiled from a number of independent resources (for details, see STAR methods). Nearly 70% of the newly identified 3′ ends were found within a distance of 100 nt from the annotations in these resources, demonstrating the suitability of our approach (Figures S1G and S1H). When critical sequencing data were re-analyzed using the more recent Ensembl Rn6 database, we obtained similar results (see STAR methods). Analysis of the poly(A) site (PAS) motifs within 150 nt of the 3′end revealed preferential usage of non-canonical PAS motifs for the longer 3′ UTRs (Figure S2A). The 3′ rapid amplification of cDNA ends (3′ RACE) performed for the *actin beta*, *stathmin 2*, and *cofilin1* transcripts on sympathetic neurons confirmed in all cases that the isoforms detected matched the 3′ ends identified by the screen (Figure S2B).

### Differential distribution of 3′ UTR isoforms in axons and cell bodies

Distinct 3′ UTRs are generated by alternative polyadenylation of the nascent mRNA, and the choice of PAS is regulated by tissue- and developmental stage-restricted factors (Gruber and Zavolan, 2019; Lianoglou et al., 2013). To investigate whether specific 3′ UTR isoforms were localized in axons or cell bodies, transcripts were divided into two categories: those present solely in cell bodies or those present also in axons (Figures S2C and S2D). We found 9,378 3′ UTR isoforms associated with 6,410 transcripts in axons (Figure S2E). On average, axonal transcripts expressed longer 3′ UTRs and a higher number of 3′ UTR isoforms than the cell body transcripts, with many axonal transcripts expressing three or more alternative 3′ UTRs (Figure 1E).

Next, we compared the relative usage of promoter-proximal and promoter-distal PASs between transcripts with multiple isoforms located either in cell bodies or axons. Transcripts containing two or more 3′ UTR isoforms were considered for further analysis (4,191 tandem pairs of 3′ UTR isoforms; Figure 1F), and the difference in log2 proximal-to-distal expression ratios of 3′ UTR isoforms between cell bodies and axons was calculated. A difference below −1 or above 1 (false discovery rate [FDR] < 0.01, Fisher’s exact count test) indicated, respectively, a distal or proximal shift of poly(A) usage in axons compared with the cell body. We found 737 isoforms (17.7% of tandem 3′ UTR isoforms) that displayed increased usage of distal PAS in axons and therefore expressed long 3′ UTR (Figure 1F, dark blue dots) and 689 transcripts (16.5% of tandem isoforms) that preferentially expressed short 3′ UTR isoforms in axons (Figure 1F, light blue dots), with high correlation between sample types (Spearman coefficients r = 0.97 for cell bodies samples and r = 0.64 for axon samples) (Figure S2F). Gene Ontology (GO) functional analysis revealed that terms associated with axon growth and energy and protein metabolisms were statistically overrepresented among axonal transcripts with shorter 3′ UTRs, whereas terms associated with more general biological pathways, such as intracellular signaling, were enriched among axonal transcripts with longer 3′ UTRs (Figure 1G). A subset of transcripts selected by applying a thresholding method (see the Figure 1F legend and STAR methods for details) displayed extreme differences in isoform usage, 63 transcripts with longer 3′ UTR and 128 transcripts with shorter 3′ UTR were either uniquely detected or expressed at very high levels in axons (Figure 1F, inset). Examples of transcripts with strikingly distinct poly(A) usage in cell bodies or axons are shown in Figures 1H, S3A, and S3B.

### An *IMPA1* isoform with a short 3′ UTR is expressed only in axons

**Figure 2:**
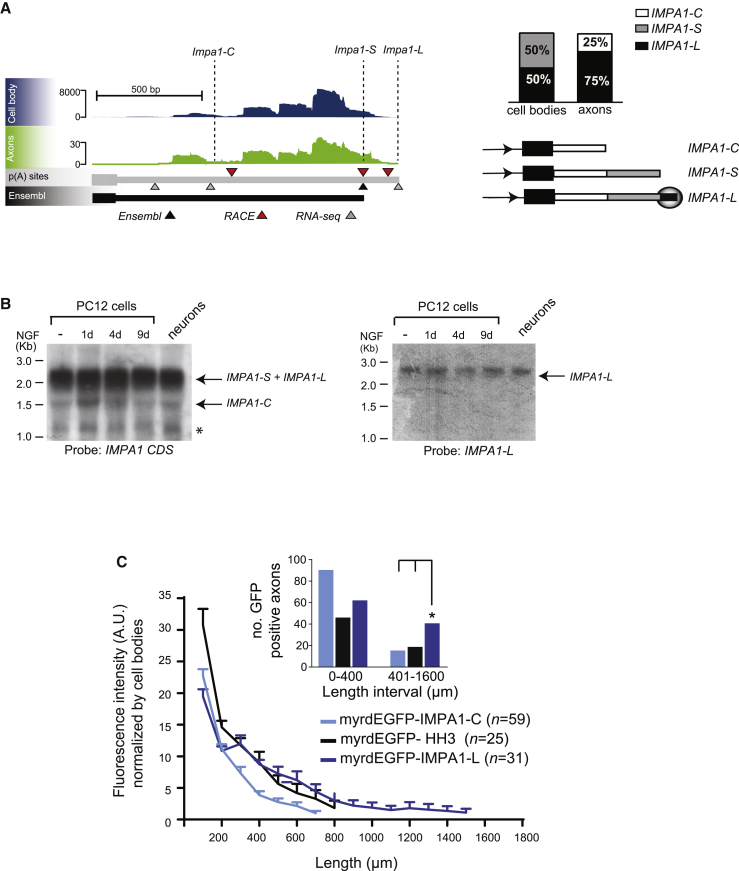
Remodeling of *IMPA1* 3′ UTR in axons (A) Left: genome browser view of *IMPA1* transcripts in axons and cell bodies transcriptomes by 3′ end RNA-seq, Ensembl annotation, and 3′ RACE (gray, black, and red arrowheads, respectively). Dashed lines indicate 3′ end of the isoforms. Right: percentage of RACE clones (top) containing different *IMPA1* 3′ UTR isoforms in axons and cell bodies (cell bodies n = 15, axons n = 12), and schematics of the three *IMPA1* 3′ UTRs (bottom). Shadowed circle indicates axonal localization signal in *IMPA1-L* 3′ UTR. (B) Northern blot analysis of RNA isolated from naive (-) or NGF-differentiated PC12 cells and sympathetic neurons using probes annealing with *IMPA1* CDS (left) or *IMPA-L* 3′ UTR (right). Note that the resolution of agarose gels and the abundance of the *IMPA1-S* isoform prevent the discrimination of *IMPA1-S* and *IMPA1-L* when using *IMPA1* CDS probe. Asterisk (^∗^) indicates a further isoform with a very short 3′ UTR detected by *IMPA1* CDS probe. Representative images from 3 independent experiments. (C) Quantitative analysis of GFP protein immunofluorescence in axons of sympathetic neurons expressing either myrdEGFP-IMPA1-L, myrdEGFP-IMPA1-C, or myrdEGFP-Histone H3 (HH3). (Inset) Distribution of GFP-positive axons at the indicated length intervals (^∗^p = 0.0108; chi-square = 6.494, df = 1). See also Figure S2.

The finding that many shorter 3′ UTR isoforms were detected solely in axons prompted us to investigate whether they may be the result of local remodeling. We previously discovered that the *IMPA1* transcript is transported to, and highly enriched in, sympathetic neuron axons and that its local translation is necessary for maintaining axon integrity (Andreassi et al., 2010). IMPA1 is the enzyme that regulates the inositol cycle and plays a key role in both the synthesis of *de novo* inositol and the recycling of inositol polyphosphates generated upon receptor activation (Resnick and Saiardi, 2008). The inositol ring is the structure upon which many intracellular messengers are built, including the calcium-releasing factor I(1,4,5)P_3_ and several membrane phosphoinositides (Di Paolo and De Camilli, 2006). Importantly, IMPA1 is one of the targets of lithium in neurons (Berridge et al., 1989) and has been implicated in the pathogenesis of bipolar disorders (Saiardi and Mudge, 2018). The 3′ end RNA-seq revealed that in sympathetic neurons, *IMPA1* expresses three isoforms bearing 3′ UTRs of different length (Figure 2A, left). Two major isoforms named *IMPA1-Short* (*IMPA1-S*, 3′ UTR 1,128 nt) and *IMPA1-Long* (*IMPA1-L*, containing an additional 120 nt axonal localization element, 3′ UTR 1,248 nt) were expressed in cell bodies and cell bodies and axons, respectively, while the third newly identified isoform carrying a much shorter 3′ UTR was detected only in axons. We named this axon-specific isoform *IMPA1-Cleaved* (*IMPA1-C*, 3′ UTR 451 nt). The distribution of *IMPA1* 3′ UTR isoforms was further confirmed by 3′ RACE on mRNA isolated either from cell bodies or distal axons of sympathetic neurons grown in compartmentalized chambers (Figure 2A, right). Northern blot analysis showed that the three isoforms were expressed in sympathetic neurons and PC12 cells (Figure 2B).

Lack of detection of short isoforms in cell bodies may be due to the fact that they are generated co-transcriptionally by alternative polyadenylation and rapidly transported to axons. To test whether localization elements present in the *IMPA1-C* 3′ UTR target the transcript to axons, we used a reporter assay based on the expression of myrdEGFP, a myristoylated and destabilized form of GFP with a very short half-life and limited intracellular diffusion (Aakalu et al., 2001). We previously demonstrated that a sequence found at the 3′end of *IMPA1-L* was necessary and sufficient to localize the transcript to axons (Andreassi et al., 2010). When sympathetic neurons were electroporated with myrdGFP-IMPA1-L, the GFP signal was clearly detected in axons up to 1,600 μm from the cell bodies. By contrast, the signal from myrdGFP-IMPA1-C was restricted to cell bodies and proximal axons (Figures 2C and S4A), indicating that similarly to *IMPA1-S* and *Histone H3* (*HH3*), the short 3′ UTRs lacking the localization element cannot target the transcript to distal axons, despite being expressed at similar levels (Figures S4B–S4D). Because *IMPA1-C* was detected only in axons by 3′ RACE, but the transport assay suggests that it cannot be localized to distal axons, we hypothesized that the short 3′ UTR of *IMPA1-C* may be generated *in situ* by *IMPA1-L* cleavage. Interestingly, several transcripts expressing shorter 3′ UTRs and potentially undergoing local remodeling were also found in dendrites of hippocampal neurons (Tushev et al., 2018), suggesting that this could be a widespread phenomenon associated with distally localized mRNAs.

### Endonucleolytic cleavage of axonal transcripts

**Figure 3:**
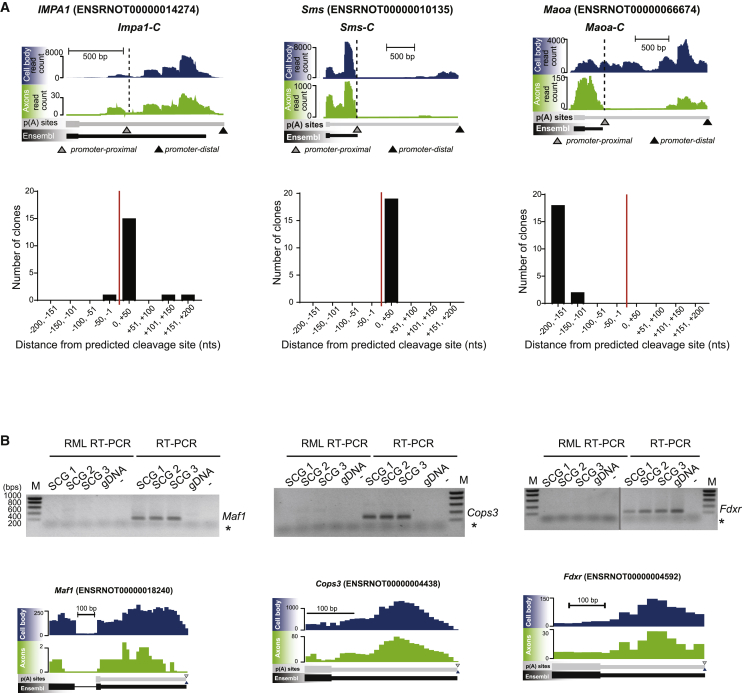
The 3′ UTRs of many transcripts are remodeled in axons (A) Top: genome browser view of *IMPA1*, *Sms*, and *Maoa* transcripts in sympathetic neuron axons and cell bodies transcriptomes by 3′ end RNA-seq and Ensembl annotation (gray and black arrowhead, respectively). Dashed lines indicate 3′ end of the short isoforms. Bottom: number of clones of cleaved *IMPA1-L*, *Sms*, and *Maoa* 3′ UTR fragments purified from axonal RNA and grouped according to distance from the predicted cleavage site (red line). Each bin represents 50 nt. (B) Absence of cleaved fragments in *Maf1*, *Cops3*, and *Fdxr* transcripts (top, left lanes). The presence of corresponding cDNAs was assessed by regular RT-PCR (top, right lanes). Gray vertical line indicates samples ran on separated gel. Asterisk (^∗^) represents primer dimers bands. Primers to amplify *Fdxr* cDNA are intra-exonic therefore they amplify also genomic DNA (gDNA). Noncontiguous lanes from the same experiment are shown side by side, as indicated by the gray line. Bottom: genome browser view of *Maf1*, *Cops3*, and *Fdxr* transcripts in axons and cell bodies transcriptomes by 3′ end RNA-seq and Ensembl annotation (gray and black arrowhead, respectively). See also Figure S3.

To explore whether the 3′ UTR of *IMPA1-L* is cleaved in axons, we used a modified RT-PCR protocol (named RNA oligonucleotide (oligo)-mediated ligation [RML] RT-PCR) that allows the amplification and cloning of 3′ UTR fragments generated by cleavage (Figure S5A). We reasoned that the predicted cleavage site would likely be in the proximity of the proximal PAS, as the 3′ UTR processing would generate an isoform expressing a shorter polyadenylated 3′ UTR. RML RT-PCR of mRNA isolated from severed axons (Figure S5B) and performed on *IMPA1*, *Sms*, and *Maoa*, two transcripts that showed an isoform expression pattern similar to *IMPA1*, revealed that most clones contained fragments corresponding to the cleaved 3′ UTR fragments (Figures 3A, S5C, and S5D). Remarkably, the fragments were stable, homogeneous in size, and mapped to precise positions relative to our predicted cleavage site, suggesting that they are not generated by 5′–3′ exonucleolytic degradation. By contrast, 3′ UTR cleavage was not detected in axonal transcripts that did not show alternative PAS usage, such as *Cops3*, *Fdxr*, and *Maf1* (Figure 3B). Thus, our findings indicate that the axonal specific short isoforms of *IMPA1*, *Maoa*, and *Sms* are generated through a process of 3′ UTR remodeling that takes place in axons.

### An Ago2 complex mediates *IMPA1-L* 3′ UTR cleavage in axons

We next sought to identify the RBPs that mediate the 3′ UTR cleavage. A potential candidate was the nuclear cleavage and polyadenylation specificity factor CPSF3 (Mandel et al., 2006). However, neither *CSPF3* mRNA nor protein was detected in axons (Figure S5E).

**Figure 4:**
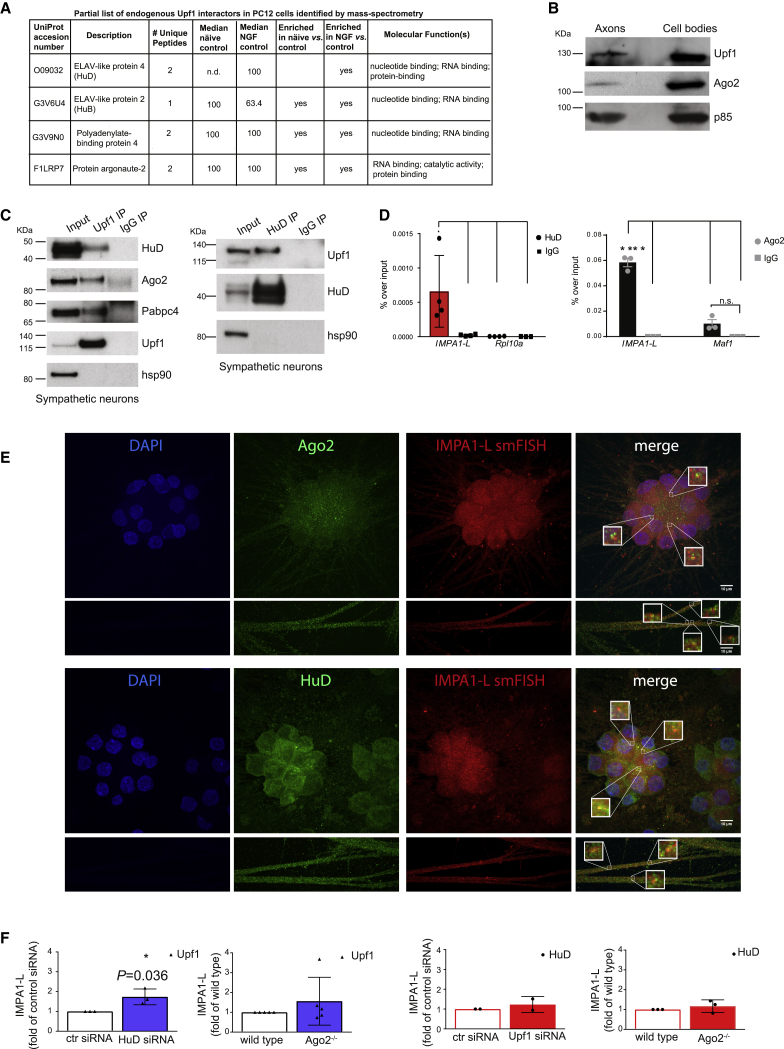
A complex containing Ago2, HuD, and Upf1 interacts with IMPA1 transcript (A) Partial list of Upf1 interactors identified in PC12 cells by mass spectrometry. n.d., not detected. (B) Western blots of Upf1, Ago2, and PI3K subunit p85 (as loading control) on axons and cell bodies of sympathetic neurons (n = 3). (C) Co-immunoprecipitation of Upf1 (left) or HuD (right) with the indicated proteins in sympathetic neurons (n = 3). (D) RNA immunoprecipitation (RIP) of *IMPA1-L*, *Rpl10a* (as negative control), or *Maf1* mRNA, with HuD (left) or Ago2 antibody (right), and normal IgG antibody in sympathetic neuron lysates. Data are means ± SEM of ΔΔCt values between antibody or immunoglobulin G (IgG) samples and respective inputs expressed as fold of inputs (^∗^p = 0.0465, paired one-tailed t test, t = 2.434, df = 3, n = 4; ^∗∗∗∗^p < 0.0001 two-way ANOVA, Sidak’s multiple comparison test t = 22.11, df = 4, n = 3). n.s., not statistically significant. (E) Immuno-FISH of *IMPA1-L* transcript with Ago2 (top) and HuD (bottom) proteins. mRNA puncta were not subject to pixel dilation. Insets- show 5X Mmagnification of boxed area. Scale bar, 10 μm. (F) RIP of *IMPA1-L* transcript with Upf1 or HuD antibodies. Experiments were performed on PC12 cells either transfected with control (siRNA), *HuD*, or *Upf1* siRNA or on wild-type (WT) or CRISPR-deleted Ago2 (*Ago2*^*−/−*^) PC12 cells. (*p = 0.036, unpaired two-tail t test, t = 3.179, df = 4; only statistically significant comparisons are indicated, n as indicated.) See also Figure S4 and Tables S1–S3.

Mass spectrometry analysis of proteins associated with polyadenylated transcripts in sympathetic neurons previously performed in our laboratory had revealed that the DNA/RNA helicase Upf1 was one of the few RBPs that interacted with axonal transcripts in response to NGF (A. Ludanyi, M.G., and A.R., unpublished data). Upf1 is part of the complex that mediates nonsense-mediated decay (NMD) of mRNA, an RNA surveillance pathway that induces rapid degradation of transcripts harboring a premature termination codon to prevent translation of truncated proteins (Kurosaki and Maquat, 2013). In addition, binding of Upf1 is enriched on longer 3′ UTRs and contributes to maintaining mRNAs in a translationally silent state (Hurt et al., 2013). Since Upf1 does not have cleavage activity and because RBPs are usually found within large multi-protein complexes, we performed mass spectrometry analysis of proteins that co-immunoprecipitated with Upf1 (Figure S6A). We identified 325 unique peptides that mapped on 72 proteins (Tables S1–S3), including known interactors of Upf1, such as Polyadenylate-binding protein 1 (Pabp1), ELAV-like protein 2, and interleukin enhancer-binding factor 2 (BioGRID:https://thebiogrid.org/111908/summary/homo-sapiens/upf1.html). Interestingly the endonuclease Ago2 and the neuron-specific HuD were among the most abundant proteins that co-immunoprecipitated with Upf1 (Figure 4A). As for most RBPs, HuD and Ago2 regulate many aspects of RNA metabolism, including alternative splicing, alternative polyadenylation, and mRNA stability and translation (Meister, 2013; Perrone-Bizzozero and Bird, 2013; Yoo et al., 2013). Both Upf1 and Ago2 were clearly detected in sympathetic neuron axons (Figures 4B and 4E), and co-immunoprecipitation experiments confirmed the interaction of Upf1 with HuD, Ago2, and the Poly(A)-binding protein cytoplasmic 4 (Pabpc4) in neurons (Figure 4C). RNA immunoprecipitation (RIP) assays performed on sympathetic neurons showed a robust interaction of Ago2, HuD, and Upf1 with *IMPA1-L* 3′ UTR (Figures 4D and S6B–S6D). We detected significantly less binding of Ago2 to *Maf1* 3′ UTR (Figure 4D), a transcript that is not predicted to undergo 3′ UTR cleavage (Figure 3B). Immuno-RNA FISH confirmed the colocalization of Ago2 and HuD with IMPA1 transcript (Figure 4E). Further insights into the mechanisms regulating the assembly of the cleavage complex on *IMPA1-L* 3′ UTR were provided by RIP experiments performed on PC12 cells lacking either HuD, Ago2, or Upf1 (Figures S6C and S6E). We found that while HuD recruitment to *IMPA1* RNA is independent of Ago2 and Upf1, Upf1 binding is regulated by both Ago2 and to a lesser extent HuD (Figure 4F), suggesting that HuD binding may represent one of the initial steps for the formation of the complex.

**Figure 5:**
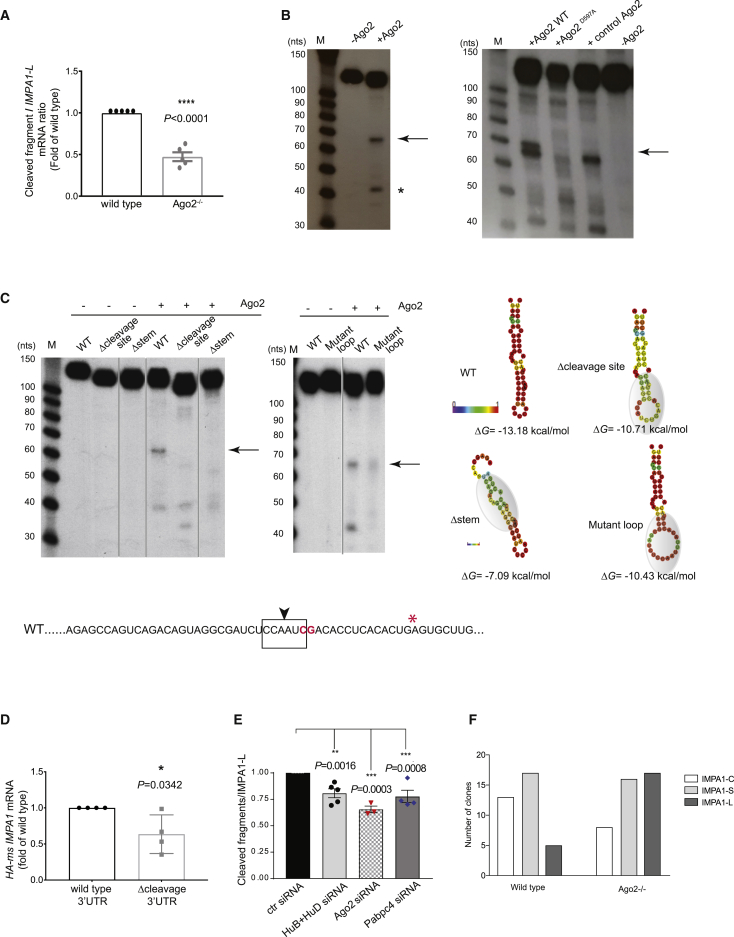
Ago2 mediates the cleavage of IMPA1 3′ UTR (A) RNA oligonucleotide (oligo)-mediated ligation (RML) of *IMPA1-L* transcript followed by qRT-PCR of WT or *Ago2*^*−/−*^ PC12 cells. Results are presented as fold over WT samples. Unpaired two-tailed t test, t = 9.912, df = 8 (n = 5). (B) Radioactive *in vitro* cleavage assay of 5′ end-labeled *IMPA1-L* RNA oligos using cytoplasmic lysates of sympathetic neurons and human recombinant Ago2 (left) or mouse recombinant WT or catalytic mutant (Ago2^D597A^) Ago2. Control Ago2 is a commercially available human WT Ago2 (right). Arrows indicate cleaved fragment, and asterisk indicates a smaller fragment, probably due to trimming of the main fragment. Irrelevant lanes have been removed (n = 3). (C) Radioactive *in vitro* cleavage assay of 5′ end-labeled WT or mutant *IMPA1-L* RNA oligos. Left: a band corresponding to the expected size of the cleaved fragment (67 nt, arrows) is detected in WT oligos while it is absent in mutants. Noncontiguous lanes from the same experiment and autoradiography blot are shown side by side, while irrelevant lanes have been removed, as indicated by the gray lines (n = 4). Right: folding predictions of WT and mutant *IMPA1-L* oligos. Color-coded probability of pairing is shown as heatmap. Δ*G* values are indicated. Shadowed area points to the effect of the mutation on the secondary structure of the oligo. (Bottom) Sequence of the WT oligo used for folding prediction. Arrowhead indicates the point of cleavage. Boxed sequence is deleted in the Δcleavage site mutant. Nucleotides in red bold are mutated in the mutant loop oligo. Asterisk points to the truncation of the Δstem mutant. (D) *HA*-ms *IMPA1* RIP in PC12 cells expressing either WT HA-*ms* IMPA1-*rat* L 3′ UTR or a mutant bearing a deletion of the cleavage site (Δcleavage-3′ UTR). Data were normalized by *c-myc* mRNA as a positive control. Unpaired two-tailed t test (n = 4). (E) *IMPA1-L* cleavage was assayed by RML qRT-PCR on RNA purified from PC12 cells transfected with the indicated siRNAs. Two-way ANOVA, Dunnett’s multiple comparison test, df = 16. (F) Number of clones bearing IMPA1-C, IMPA1-S, or IMPA1-L 3′ UTR in WT and *Ago2*^*−/−*^ PC12 cells. Thirty-five and 41 randomly selected clones obtained by 3′ RACE from WT and *Ago2*^*−/−*^ cells were sequenced. p = 0.0255, chi-square test, df = 2. All data in this figure are presented as mean ± SEM. Tests are indicated in the legend and p values in the figure. See also Figure S5.

Ago2 is the only member of the Argonaute family of proteins with endonuclease activity and is known to bind preferentially to long 3′ UTRs (Meister, 2013). To investigate whether Ago2 was the endonuclease responsible for the cleavage of *IMPA1* 3′ UTR, PC12 cells lacking Ago2 were generated by CRISPR (Figure S6E). In the absence of Ago2, the cleavage of IMPA1 3′ UTR was significantly reduced as assessed by RML qRT-PCR (Figure 5A), indicating that Ago2 is one of the endonucleases responsible for the 3′ UTR processing. To further assess the contribution of Ago2 to 3′ UTR remodeling, we designed an *in vitro* cleavage assay. Recombinant Ago2 was incubated with a 5′ end-labeled RNA oligo encompassing the predicted cleavage site and cytoplasmic lysates of sympathetic neurons. A stable fragment of the expected size was detected (64 nt; Figures 5B and S6F), together with a smaller fragment probably generated by the trimming of the primary cleaved fragment (Cheloufi et al., 2010). When the cleavage assay was performed using a recombinant catalytic mutant Ago2 bearing a mutation of aspartate^597^ to alanine, which abolished the endonuclease activity (Liu et al., 2004), *IMPA1* 3′ UTR cleavage was greatly reduced (Figures 5B, S6G, and S6H). Moreover, mutations of the cleavage site identified with the RML RT-PCR assay abolished Ago2-dependent cleavage (Figure 5C, Δcleavage site), while decreasing the binding of Ago2 to *IMPA1-L* 3′ UTR (Figure 5D).

Ago2 is a double-strand (ds)RNA endonuclease that typically cuts through microRNA (miRNA) paired to target mRNA (Meister, 2013). However, Ago2 can also cleave miRNA precursors and mimetics by recognizing stem-loop structures (Cheloufi et al., 2010; Harwig et al., 2017). When *IMPA1-L* 3′ UTR was run through the RNA folding prediction software RNAfold (Gruber et al., 2008), the sequence surrounding the predicted cleavage site formed a stable stem-loop (Figures 5C, wild type [WT], and S6I). To test whether Ago2-dependent cleavage of *IMPA1-L* 3′ UTRs may occur through the binding and cleavage of a dsRNA structure, we synthesized oligos with either an impaired stem structure (Δstem) or an enlarged loop (mutant loop). Ago2-dependent cleavage of these mutants was virtually undetectable (Figure 5C), confirming that the stem-loop structure surrounding the cleavage site is necessary for Ago2 cleavage of *IMPA1*. It should be noted that in the Δstem mutant oligo the sequence surrounding the cleavage site is intact, indicating that Ago2 cleavage of *IMPA1-L* is not dependent on miRNA potentially targeting the cleavage site.

The cleavage assay was performed in the presence of neuronal cytoplasmic lysates, and it is expected that additional RBPs and/or co-factors are required for the Ago2-dependent cleavage reaction and the stabilization of the cleaved fragments. Indeed, silencing of either *Ago2*, *HuD/B*, or *Pabpc4* in PC12 cells decreased the cleavage of endogenous *IMPA1-L* 3′ UTR consistent with the levels of small interfering RNA (siRNA)-mediated silencing for each molecule (Figures 5E and S6J). 3′ RACE of IMPA1 clones performed in either WT or in PC12 cells lacking Ago2 confirmed that in the absence of Ago2, the number of clones expressing the cleaved 3′ UTR (*IMPA1-C*) was significantly lower, whereas the number of clones expressing *IMPA1-L* increased (Figure 5F).

Taken together, these data indicate that a protein complex containing HuD and Ago2 cleaves the 3′ UTR of a transcript localized in axons of sympathetic neurons. They also show that the 3′ UTR fragment resulting from the cleavage is stable and unlikely to be a byproduct of RNA degradation.

### Axonal remodeling of *IMPA1-L* 3′ UTR increases *IMPA1* mRNA translation and is necessary to maintain axon integrity

**Figure 6:**
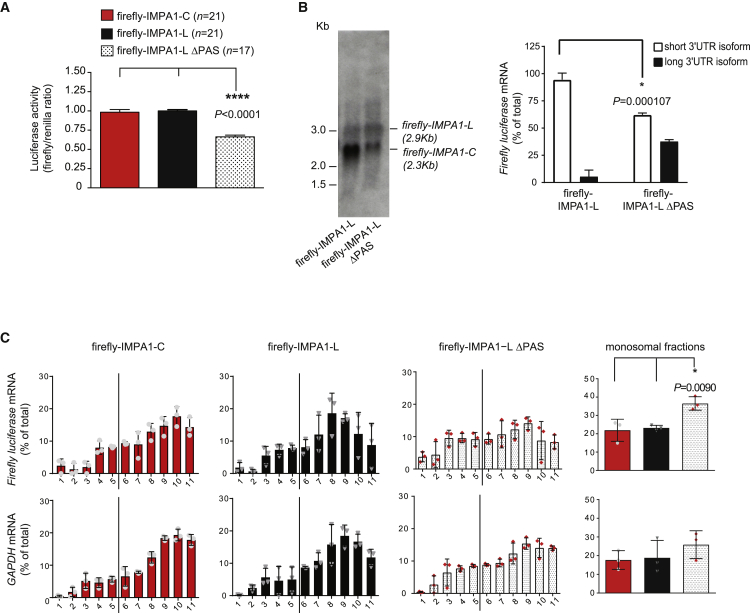
Mutation of IMPA1 proximal PAS sites decreases translation (A) Luciferase assay of PC12 cells transfected with the indicated firefly luciferase vectors and renilla luciferase, as indicated. One-way ANOVA, Tukey’s post hoc test, at least 4 independent experiments, *F* = 47.66, df = 58. (B) Left: firefly luciferase northern blotting of PC12 cells transfected with either Firefly-IMPA1-L or Firefly- IMPA1-L ΔPAS expression vectors. Irrelevant lanes have been removed. Right: quantitative analysis of *Firefly luciferase* levels. t test Holm-Sidak method, df = 4 (n = 3). (C) Lysates of PC12 cells transfected with the indicated Firefly-IMPA1 3′ UTR vectors were separated by polysomal fractionation, RNA was isolated from each fraction and subjected to northern analysis using ^32^P-labeled probes to detect *Firefly* (top) or *GAPDH* (bottom) transcripts. Bands were captured using a phosphorimager and quantified using ImageQuant v.5.2. Vertical black bar indicates separation between monosomal and polysomal fractions. (Right) Graphs of the amount of mRNA in cumulative monosomal fractions for lysates transfected with indicated vectors. One-way ANOVA, Tukey’s post hoc test, df = 6 (n = 3). All data in this figure are presented as mean ± SEM. Tests are indicated in the legend and significant p values in the figure. See also Figure S6.

We recently demonstrated that the 3′ UTR can determine whether a mRNA will be translated in neurons or will function as a non-coding transcript (Crerar et al., 2019). Moreover, 3′ UTR length correlates with translation levels in many cell types (Nam et al., 2016; Sandberg et al., 2008) including neurons (Blair et al., 2017; Flavell et al., 2008). Thus, we reasoned that 3′ UTR cleavage might regulate protein synthesis in axons. The isoform generated by the shortening of the 3′ UTR (*IMPA1-C*) was polyadenylated as efficiently as *IMPA 1-L* (Figure S7A). To assess the translation efficiency of the different *IMPA1* isoforms, we generated firefly luciferase vectors carrying *IMPA1-L* or *IMPA1-C* 3′ UTR. The results of the luciferase assays demonstrated that the *IMPA1-C* 3′ UTR promoted translation at levels similar to the *IMPA1-L* 3′ UTR (Figure 6A). Noticeably, northern blot analysis showed that firefly-IMPA1-L was able to generate a transcript of the size of *IMPA1-C* (Figure 6B). Mutation of *IMPA1-L* proximal PASs (firefly-IMPA1-L ΔPAS) decreased the levels of the shorter transcript generated from *IMPA-L* (Figure 6B) and decreased translation (Figure 6A) without affecting mRNA stability (Figure S7B). Polysomal fractionation confirmed a substantial shift of *firefly-IMPA1-L ΔPAS* toward the lighter, monosome-rich fractions that are normally associated with lower levels of translation, whereas the wild-type *firefly-IMPA1-L* (that produces high amounts of a shorter transcript of a size similar to *IMPA1-C*) and *firefly-IMPA1-C* preferentially co-sedimented with the polysome-enriched fractions (Figures 6C and S7C). Importantly, all isoforms were efficiently polyadenylated (Figures S7A and S7D).

**Figure 7:**
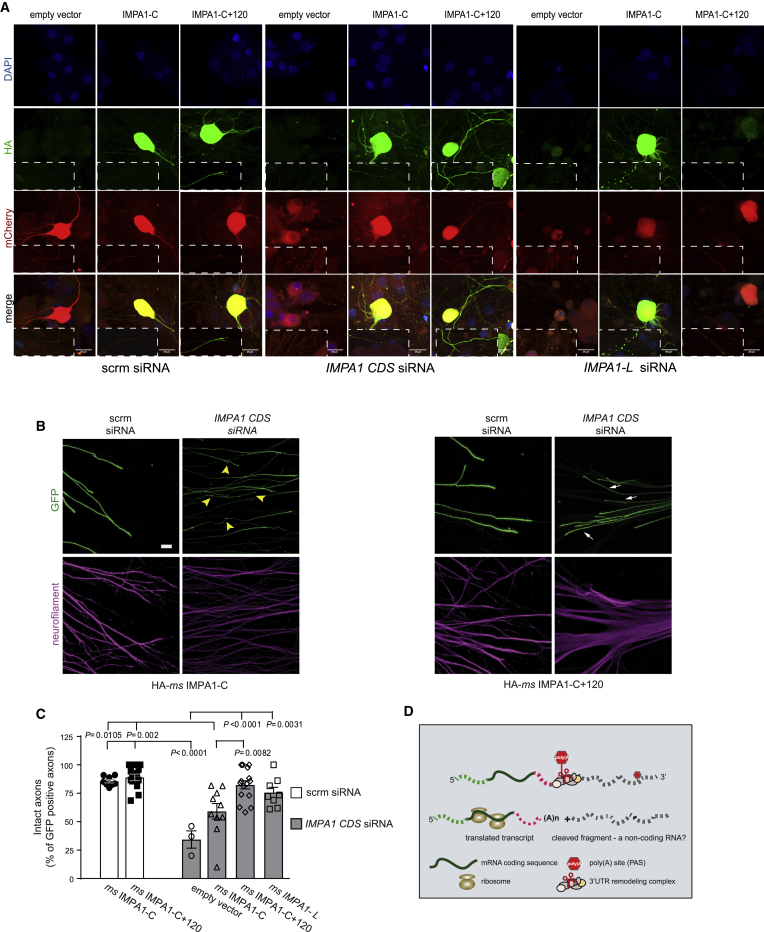
*IMPA1-C* rescues axonal degeneration when targeted distally (A) Immunostaining of sympathetic neurons and axons transfected with the indicated siRNAs and the mouse IMPA1 HA-tagged vectors. siRNAs: *scrm*, scrambled; *IMPA1 CDS*, targeting IMPA1 coding sequence; *IMPA1-L*, targeting the unique 120 nt of *IMPA1-L* 3′ UTR. Vectors: control, empty vector; IMPA1-C, expressing HA-tagged *ms* IMPA1-rat C; IMPA1-C+120, carrying also the 120 nt axonal localization element. Neurons were stained with DAPI, anti-HA, and anti-mCherry antibodies. Scale bar, 25 μm. (B) Representative images of superior cervical ganglia explants electroporated with scrambled siRNA or *IMPA1 CDS* siRNA, in the presence of HA-*ms* IMPA1-C (left) or HA*-ms* IMPA1-C+120, and GFP (right). Arrows point to healthy, intact axon bundles and arrowheads to degenerating axon bundles with characteristic beads-on-string appearance. Scale bar, 75 μm. (C) Quantitative analysis of the data shown in (A), presented as mean ± SEM. One-way ANOVA, Tukey’s multi-comparison test, *F* = 11.46, df = 104 (only statistically significant comparisons are indicated). (D) Schematic representation summarizing the cleavage and remodeling of 3′ UTR in axons. See also Figure S7.

We previously found that local synthesis of *IMPA1* in axons is necessary to maintain sympathetic axon integrity (Andreassi et al., 2010). To investigate whether *IMPA1-C* was sufficient to rescue axon degeneration induced by *IMPA1* silencing, rescue vectors containing hemagglutinin (HA)-tagged mouse *IMPA1* flanked by the rat 3′ UTR from either *IMPA1-L* or *IMPA1-C* transcripts (HA-*ms* IMPA1-L or -C) were co-transfected with siRNA targeting the coding region of rat *IMPA1* (*IMPA1-CDS* siRNA; Figure S7E). Transfection of HA-*ms* IMPA1-L, an isoform that is transported in axons, rescued axon degeneration induced by IMPA1 silencing (Figures 7A–7C). Conversely, HA-*ms* IMPA1-C, which cannot be transported to axons, did not significantly rescue the axon degeneration induced by *IMPA1* silencing. The small rescue provided by HA-*ms* IMPA1-C is probably a consequence of freely diffusible inositol from the cell bodies that may compensate for the lack of IMPA1 activity in axons. In fact, the embryonic lethality of mice lacking IMPA1 is reversed by inositol supplementation to the pregnant mothers (Cryns et al., 2008). When the 120 nt localization element was added to *IMPA1-C* to force the transport of the transcript in axons (HA-*ms* IMPA1-C+120), axonal survival in neurons lacking IMPA1 was fully preserved (Figures 7A–7C and S7F). These findings confirm that *IMPA1-C* 3′ UTR lacks the localization element necessary for axonal transport (Figure 2C). They also show that when forcibly targeted to axons, *IMPA1-C* is as efficient as *IMPA1-L* in promoting axon integrity. This suggests that the axonal cleavage of long 3′ UTRs may be critical for mRNA translation and thereby for the physiological function of the resultant protein.

## Discussion

The untranslated regions of mRNAs play a critical role in the regulation of transcript localization and translation in virtually all mammalian tissues. Global mapping of 3′ end regions indicated that ∼75% of mammalian genes contain more than one PAS, giving rise to multiple 3′ UTRs (Gruber and Zavolan, 2019; Proudfoot, 2011; Tian and Manley, 2013). Noticeably, PAS usage and 3′ UTR length vary remarkably between mammalian tissues. For example, transcripts in the nervous system are characterized by significantly longer 3′ UTRs compared with blood and testis (Miura et al., 2013). A switch in PAS usage has been observed in a variety of biological processes. Proximal PASs are preferentially used in proliferating cells, in response to inflammation, or in cancerous cells to generate transcripts with shorter 3′ UTRs (Blair et al., 2017; Flavell et al., 2008; Mayr and Bartel, 2009; Sandberg et al., 2008). Conversely, tissue differentiation is often associated with a switch from proximal-to-distal PAS and 3′ UTR lengthening.

In neurons, 3′ UTR lengthening of a magnitude greater than in any other tissue takes place during development, with many transcripts bearing unusually long 3′ UTRs (Miura et al., 2013; Tushev et al., 2018) and alternative last exons (ALEs) (Taliaferro et al., 2016). We recently demonstrated that in sympathetic neurons, 3′ UTR length can even determine whether a transcript will be translated or will function in a non-coding manner by interacting with the transmembrane NGF receptor TrkA (Crerar et al., 2019). Alternative 3′ UTR usage is observed also at the subcellular level as our 3′ end RNA-seq analysis revealed preferential use of distal PAS and 3′ UTR lengthening for transcripts localized to axons when compared with cell bodies (Figure 1C). Moreover, the number of PAS motifs increased with 3′ UTR length (Figure S2A), and a higher number of 3′ UTR isoforms per transcript are expressed in axons (Figure 1E). The generation of multiple isoforms for the axonal transcriptome may play an important role in determining the localization of transcripts through the inclusion of specific localization elements and can contribute to fine-tuning protein synthesis in response to environmental conditions.

Once transcripts have reached their peripheral destination, they are locally translated in response to extrinsic stimuli including synaptic activation, neurotrophic factors, and axon guidance cues (Andreassi et al., 2018; Biever et al., 2019; Holt et al., 2019). In hippocampal neurons, a switch to proximal PAS usage and shortening of 3′ UTRs was observed in genes that become transcriptionally activated in response to depolarization, perhaps indicating that a shorter 3′ UTR enhances translation under these conditions (Flavell et al., 2008). Accordingly, we found 689 isoforms with shorter 3′ UTRs that were highly enriched in sympathetic neuron axons compared with cell bodies (Figure 1F). Although in most instances these isoforms were detected also in cell bodies (albeit at much lower levels), at least 128 axonal transcripts with a shorter 3′ UTR were virtually absent in cell bodies.

PAS choice has been thought to occur exclusively in the nucleus, where transcriptional elongation is coupled with 5′ end capping, splicing of pre-mRNA, cleavage, and polyadenylation of the 3′ end (Tian and Manley, 2013). However, our findings indicate that at least for some transcripts, a remodeling of the 3′ UTR takes place at the site of protein synthesis. RACE analysis of the *IMPA1* transcript revealed that an isoform bearing a shorter 3′ UTR (*IMPA1-C*, Figure 2A) was localized in distal axons despite lacking the localization element necessary for axonal targeting (Figure 2C). *IMPA-1-C* is polyadenylated (Figure S7A) and efficiently translated (Figure 6A). When *IMPA1-C* was forcibly transported and expressed in axons, it was sufficient to rescue the axonal degeneration induced by *IMPA1* silencing (Figures 7A–7C). Importantly, we found that the long 3′ UTR of *IMPA1* undergoes cleavage in axons, generating *IMPA1-C* and a 3′ UTR fragment that was detected by 5′ end RNA ligation (Figure 3A). These findings support a model by which *IMPA1-L* is transcribed in cell bodies and then targeted to axons where the long 3′ UTR is remodeled into *IMPA1-C* and translated, possibly in response to local cues (Figure 7D). The cleavage of 3′ UTRs is not limited to *IMPA1* and was demonstrated for at least two other axonal transcripts, *Sms* and *Maoa* (Figure 3A). Importantly, a similar mechanism was demonstrated in bacteria, where cleavage of the 3′ UTR of the stress response mRNA *cpxQ* generates a coding mRNA that is translated, and a non-coding, 3′ UTR-derived fragment that mediates a number of cellular functions, including the maintenance of membrane potential (Chao and Vogel, 2016). More recently, Tushev et al. (2018) demonstrated that in hippocampal neurons numerous dendritic transcripts may undergo local remodeling in response to synaptic activation. Thus, axonal 3′ UTR remodeling may represent a general mechanism by which localized mRNA transcripts are translated in cells.

How is 3′ UTR remodeling regulated? We found that a complex containing the endonuclease Ago2 mediates *IMPA1* 3′ UTR cleavage *in vitro* (Figure 5B) and in intact cells (Figures 5A, 5E, and 5F). Our data also suggest that a specific stem-loop structure around the cleavage site is required for efficient endonuclease activity by Ago2 (Figure 5C). A recent study has shown that maturation of miRNA precursors takes place in dendrites in response to depolarization (Sambandan et al., 2017). The finding that Ago2 and Pabpc4 are part of the 3′ UTR remodeling complex (Figures 4C and 5E) suggests a potential cross-talk between local 3′ UTR processing and the miRNA machinery. Although NGF-dependent regulation of Ago2 has not been tested, HuD has been shown to mediate some downstream signaling of NGF (Fujiwara et al., 2006) and to interact with numerous neurotrophic factors mRNAs (Lim and Alkon, 2012). Given that our data indicate that HuD may represent the key step for the assembly of the cleavage complex, it will be interesting to investigate the role of NGF in Ago2-mediated remodeling of 3′ UTRs.

Remarkably, our cleavage assay demonstrated that in addition to mRNAs with shorter 3′ UTR isoforms, the remodeling generates stable 3′ UTR fragments. Widespread endonucleolytic post-transcriptional processing independent of miRNA targeting has been reported in mammalian cells (Karginov et al., 2010), and stable uncapped 3′ UTR fragments have been found in the human transcriptome (Malka et al., 2017). 3′ UTRs can be expressed independently of their coding sequence and small peptides synthesized from the 3′ UTR of genes were detected in aging dopaminergic neurons (Sudmant et al., 2018). Mercer et al. (2011) also demonstrated that in mouse, 3′ UTR-derived RNAs are widely expressed in a tissue- and cell-specific manner. It should be noted that genome-wide analysis of RNA polymerase II binding revealed a lack of occupancy at these sites, indicating that they are not generated by transcriptional events originated from promoters located within the 3′ UTRs (Mercer et al., 2011). Even more excitingly, differential expression of 3′ UTRs and coding regions were observed for many genes expressed in the developing mouse brain (Kocabas et al., 2015). Although the function of 3′ UTR-derived RNAs remains unknown, a recent study indicated that a 3′ UTR fragment generated by cytoplasmic splicing of the transcription factor *xbp-1* contributes to axon regeneration in *Caenorhabditis elegans* (Liu et al., 2020). Here, we provide evidence for the mechanism that mediates the cleavage of 3′ UTRs in rat sympathetic neurons generating coding isoforms with remodeled 3′ UTRs and a new class of 3′ UTR fragments. Therefore, our findings suggest that mRNA transcripts may simultaneously have coding-dependent and coding-independent functions, adding a remarkable layer of complexity to the regulation of gene expression.

## STAR★methods

### Key Resources Table

**Table:** 

REAGENT or RESOURCE	SOURCE	IDENTIFIER
**Antibodies**		

rabbit Anti-Ago2	Abcam	Cat#ab186733; RRID:AB_2713978
rabbit Anti-GFP	Abcam	Cat#ab655; RRID:AB_305562
Rabbit HA	CST	Cat#3724; RRID:AB_1549585
mouse HuD	Santa Cruz	Cat#Sc-28299; RRID:AB_627765
goat HuD	Santa Cruz	Cat#sc-5979; RRID:AB_2101220
mouse Anti CPSF3	Santa Cruz	Cat#sc-393001
mouse anti-mCherry	Abcam	Cat#ab125096; RRID:AB_11133266
chicken Anti-neurofilament	Abcam	Cat#ab4680; RRID:AB_304560
rabbit Anti-neurofilament	Sigma	Cat#N4142; RRID:AB_477272
mouse Pabpc4	R&D	N/A
rabbit PI3 kinase p85	Upstate	Cat#06–497; RRID:AB_310141
Goat Hsp90	Santa cruz	Cat# sc-1055; RRID:AB_2121400
Rabbit IMPA 1	Abcam	Cat#ab184165
mouse anti-alpha tubulin	Sigma	Cat#T9026; RRID:AB_477593
rabbit Anti-Upf1	Millipore	Cat#07-1014; RRID:AB_1977460

**Bacterial and virus strains**		

*E. coli* HST08 strain Stellar Competent Cells	Takara	Cat# 636763
XL10-Gold ultracompetent cells	Takara	Cat# 210518

**Chemicals, peptides, and recombinant proteins**		

human recombinant Ago2	Active Motif	Cat# 31486
mouse wild type Ago2	This paper	n/a
mouse catalytic dead (CD) Ago2	This paper	n/a

**Critical commercial assays**		

ScriptSeq	Illumina	Cat#SSV21106
PureLink® RNA Micro Scale Kit	ThermoFisher Scientific	Cat# 12183016
Poly(A) tail length assay kit	USB	Cat#76455
SMART RACE cDNA Amplification Kit	Takara	Cat#634858
Dual-Glo® Luciferase Assay System	Promega	Cat# E2920

**Deposited data**		

RNaseq data	This paper	GSE160025
Mass spec data	This paper	PXD023586
polyadenylation atlas	polyA site annotationPolyA_db Ensembl Rn6, RefSeq Rn5 and Rn6	https://doi.org/10.1093/nar/gkl870https://doi.org/10.1101/gr.202432.115

**Experimental models: cell lines**		

PC12 cells	ATCC	Cat#CRL-1721

**Experimental models: organisms/strains**		

primary sympathetic neurons from P0/P1 Sprague Dawley rats	This paper	n/a

**Oligonucleotides**		

List of all primers and oligonucleotides	See Table S4	n/a

**Recombinant DNA**		

myrdEGFP- IMPA1-C or -L or HH3	See Cloning section of STAR methods	n/a
HA-ms IMPA1-C or C+120 or Δcleavage site	See Cloning section of STAR methods	n/a
Firefly-IMPA1-C or L or LΔPAS	See Cloning section of STAR methods	n/a
mCherry expression vector	Clontech	n/a

**Software and algorithms**		

source code	This paper	GitHub: https://github.com/RLuisier/my3UTRs
ImageJ	(Schneider et al., 2012)	https://imagej.nih.gov/ij/
RNAfold Web Sever	http://rna.tbi.univie.ac.at//cgi-bin/RNAWebSuite/RNAfold.cgi	n/a

### Resource availability

#### Lead contact

Further information and requests for resources and reagents should be directed to and will be fulfilled by the Lead Contact, Antonella Riccio a.riccio@ucl.ac.uk

#### Materials availability

Further requests for resources and reagents should be directed to and will be fulfilled by the Lead Contact, Antonella Riccio a.riccio@ucl.ac.uk. Some materials may be available from the Lead Contact with a completed Materials Transfer Agreement.

#### Data and code availability

The accession number for the sequencing data (fastq files) generated in this study is GEO: GSE160025 (https://www.ncbi.nlm.nih.gov/geo/query/acc.cgi?acc=GSE160025). All the custom code (which is not a software but rather a compilation of R, Python and Bash codes which perform the re-annotation of the 3′ end and the down-stream analysis; preparation of the figures) can be freely accessed on GitHub : https://github.com/RLuisier/my3UTRs. This repository also contains the output of the pipeline (gtf annotation file and count matrix).

The mass spectrometry proteomics data have been deposited to the ProteomeXchange Consortium PRIDE: PXD023586.

Raw data from Figures 1, 2, 3, 4, 5, 6, and 7 and S1, S2, and S4–S7 have been deposited to Mendeley Data: https://data.mendeley.com/datasets/bwr4rmcpwt/draft?a = 090bfc69-6687-40e5-a093-85fed39f28 de.

### Experimental model and subject details

#### Primary cultures and ethical approval

All animal studies were approved by the Institutional Animal Care and Use Committees at University College London. Superior cervical ganglia (SCG) were dissected from post-natal day 1 (P1) Sprague Dawley rats of both sex and used for explants or enzymatically dissociated and plated in dishes or in compartmentalized chambers, as previously described (Andreassi et al., 2010). SCG explants were cultured on poly(D)lysine-laminin for 9-10 days before surgical removal of cell bodies. Cultures were maintained in DMEM containing 10% Fetal Bovine Serum, 5% Horse Serum (Hyclone), 2mM glutamine, 1% antibiotics, NGF (concentrations as specified in the relevant Methods details section) at 37°C, 10% CO_2_.

#### Cell lines

PC12 cells (purchased from ATCC) were maintained in DMEM containing 10% Fetal Bovine Serum, 5% Horse Serum (Hyclone), 2mM glutamine, at 37°C, 10% CO_2_. To induce cell differentiation serum concentration was reduced to 0.5% FBS and 0.25% HS and 50ng/mL NGF was added for the indicated time. Cell lines were routinely tested (negatively) for mycoplasma.

### Methods details

#### Reagents

Cell culture reagents, molecular biology reagents and kits were purchased from Thermo Fisher Scientific and all other chemicals from Sigma, unless stated otherwise.

#### Cell transfection and CRISPR/Cas9 mutagenesis

For transient silencing and overexpression studies, PC12 cells were transfected with Lipofectamine2000 in OptiMEM according to the manufacturer’s instructions. To generate *Ago*_*2*_^*−/−*^ clones, low-passage PC12 cells were co-transfected with 50pmol of pre-annealed Ago2-specific crRNA oligos and *trans*-activating crRNA (purchased from Integrated DNA Technologies) and 31pmol of Alt-R® S.p. Cas9 Nuclease V3 (Integrated DNA Technologies) using Neon Transfection System (ThermoFisher Scientific) as per manufacturer’s protocol. Single clones were isolated by limiting dilution in 96-well plates and confirmed by visual examination of the plates at first plating. The clonal cell lines were amplified before screening by western blot for absence of Ago2 protein. crRNA sequence, antibodies and probing conditions are described in Table S4.

#### RNA isolation, reverse transcription, linear amplification and 3′ end RNA-seq

To ensure that the axons were free of cell bodies, prior to each experiment axon compartments were incubated with Hoechst 33342 (10 μg/mL in PBS for 20 min at 37°C) and observed under an inverted fluorescent microscope. Cultures showing cell nuclei in the axon compartments or leakage of the dye in the central compartment were discarded. Total axonal and cell bodies RNA was purified from the lateral compartments of 52 or 36 chambers and the central compartment of 7 or 6 chambers respectively, obtained from 3 or more independent cultures. Total RNA was isolated using PureLink® RNA Micro Scale Kit, according to the manufacturer’s instructions with minor modifications. Briefly, axons and cell bodies were collected from chambers using lysis buffer (300 μL) containing 10% β−mercaptoethanol. Total mRNA bound to the columns was washed and eluted twice in elution buffer (12 μL). Aliquots of each sample were reverse transcribed in a 20 μL reaction volume containing random hexamer mix and 50U SuperScript III Reverse Transcriptase at 50°C for 1 hr. To check the quality of samples and the absence of cell bodies contamination in axon samples, first-strand cDNAs (5 μL) were PCR amplified in a 25 μL PCR reaction containing actin beta or histone H4 specific primers (0.20 μM), dNTPs (200nM) and Go Taq polymerase (1.25U, Promega). Primer sequences and PCR conditions are provided in Table S4.

For mRNA linear amplification, samples were purified as described above, concentrated by speed-vacuum centrifugation to 1 μL (axons) or 5 μL (cell bodies) volume, and used for two rounds of linear amplification as previously described (Baugh et al., 2001). The volume of the first-strand reaction for the axons was scaled down to 5 μL. After the second round of amplification contaminant cDNA was digested by treating the samples with RNase-free DNase (2U, Epicenter). Performance of the samples was tested by RT-PCR. Linear amplified aRNA from cell bodies and axon samples (2 biological replicates each) was used to prepare RNASeq libraries using the strand-specific ScriptSeq protocol (Illumina). Paired-end sequencing (2x 150bp) of four indexed libraries was performed on the Illumina HiSeq2000 platform, generating in excess of 80M mappable reads per sample. Library preparation and sequencing were performed at the Liverpool Centre for Genomic Research (CGR, https://www.liverpool.ac.uk/genomic-research/). Statistics of the sequencing are shown in Table S5.

#### Inference of 3′ UTR isoforms from 3′ end RNA-seq

Paired-end stranded RNA-seq reads of 150 bp were mapped to the reference rat genome (UCSC, rn5) using TopHat2 (Kim et al., 2013) allowing up to 20 multi-alignments and 2 read mismatches. The extension of the rat 3′ UTR isoform annotation was performed in two steps: 1) by identifying the longest 3′ UTR, and 2) within this longest 3′ UTR, by identifying alternative 3′ UTR isoforms. To find the longest 3′ UTR, nucleotide-level stranded coverage was first obtained for axonal and cell body samples using genomecov from the BEDTools suite (Quinlan, 2014; Quinlan and Hall, 2010). Continuously transcribed regions were next identified using a sliding window across the genome requiring a minimum coverage of 7 reads in more than 80 positions per window of 100 bp; neighboring regions separated by low mappable regions were merged as described in Miura et al. (2013). Expressed fragments were associated with matching strand overlapping 3′ UTR using Ensembl Rn5 version 78 (v78) (Flicek et al., 2014). Isolated expressed fragments that did not overlap with any feature were associated with the closest 3′ UTR if (1) the 3′ UTR was < 10kb and (2) there were no intervening annotations. We filtered assigned expressed fragments to exclude potential intragenic transcription, overlapping transcripts, and retained introns as described in Miura et al. (2013). If the expressed sequence continued beyond the end of the annotated 3′ UTR, we took the sequence as a new 3′ end. We also repeated the analysis using Ensembl Rn6 database and obtained similar results to the annotation performed using Ensembl Rn5.

The workflow used to generate input samples for the 3′ end RNA-seq data includes two rounds of linear mRNA amplification as described in Baugh et al. (2001), which leads to accumulation of the reads at the 3′ end of the transcript. Thus, a marked change in the level of coverage in the 3′ to 5′ end direction is expected to occur at the boundaries of alternative 3′ ends within longest annotated 3′ UTR (the read coverage which arises from such experiment looks like the coverage depicted on Figure S2B). To identify alternative 3′ UTR isoforms we smoothed base-level read coverage along longest 3′ UTR using a running median of 150 nt width (corresponds to read length). We then used the R package Segmentor3IsBack (Cleynen et al., 2014) to identify positions of change-point along the 3′ UTR that are hypothesized to coincide with 3′ ends. The algorithm models the nucleotide read coverage using a negative binomial distribution to first estimate the number of segments via a penalized likelihood criterion (we imposed an upper boundary of 10 segments) and then identifies change-points along the coverage by determining the global maximum of the log-likelihood of a piece-wise constant model. We applied the algorithm to the raw coverage and log2-scaled coverage of both cell body and axon-derived samples. We then merged all 4 annotations (cell body and axon samples, linear and log scale) and clustered 3′ end located within 50 nts distance, selecting the most promoter-distal annotation. We searched the −100 nts to +50 nts region surrounding the 3′ end termini of Ensembl annotated and newly annotated 3′ UTR isoforms for 12 canonical and non-canonical PAS motifs (AATACA, ATTAAA, TATAAA, AATATA, AATAGA, AGTAAA, AATGAA, ACTAAA, CATAAA, GATAAA, AAGAAA, and AATAAA) listed in PolyA_db (Lee et al., 2007) using the matchPattern function from the Biostrings R package: https://bioconductor.org/packages/Biostrings. We tested for the statistical enrichment of the PAS motifs in 3′ UTR isoforms using the Fisher’s exact test. A polyadenylation sites atlas was combined from the following sources: 1) poly(A) site annotation (Gruber et al., 2016) build using 3′ end sequencing libraries in human and mouse, lifted from hg19/mm10 to Rn5 using python library CrossMap (Zhao et al., 2014); 2) 3′ end sequencing libraries from rat brain and testes (31) (Derti et al., 2012); 3) 3′end annotation in Ensembl Rn6, RefSeq Rn5 and Rn6, and XenoRefSeq; 4) polyadenylation sites annotations from PolyA_DB (Lee et al., 2007) and APADB (Müller et al., 2014). We next compared the percentage of newly annotated 3′ ends recovered from each source and from the compiled polyadenylation site atlas at several intervals from novel 3′ ends.

#### 3′ UTR isoform quantification and identification of transcripts localized to axons

The number of reads mapped to −500 nts terminal region of each 3′ UTR isoform was used to calculate the expression levels. The density of mapped reads in −500 nts terminal region of 3′ UTR isoforms is bimodal, with a low-density peak probably corresponding to background transcription, i.e., 3′ UTR isoforms of low abundance or 3′ UTR isoforms to which reads were spuriously mapped, and a high-density peak corresponding to expressed 3′ UTR isoforms. In order to identify 3′ UTR isoforms expressed in axons and cell body, a two-component Gaussian mixture was fitted to the data using the R package mclust: https://mclust-org.github.io/mclust/. An isoform was called expressed if in both replicates there were less than 5% chance of belonging to the background category or if in at least one replicate there was more than 10% chance of belonging to the expressed category (Barford et al., 2017).

#### Differential 3′ UTR isoforms expression analysis

We focused the analysis on 4,191 tandem pairs of 3′ UTR isoforms expressed in the cell body and/or in axonal samples. To identify transcripts displaying a change in the 3′ UTR isoform usage between axon and cell body samples, we scored the differences in promoter-proximal to promoter-distal poly(A) site usage:

$$S_{1}=log_{2}{\left(\frac{I_{proximal}}{I_{distal}}\right)}_{CB}-log_{2}{\left(\frac{I_{proximal}}{I_{distal}}\right)}_{Axons}$$

$$S_{2}={\frac{I_{proximal}}{I_{proximal}+I_{distal}}}_{CB}-{\frac{I_{proximal}}{I_{proximal}+I_{distal}}}_{Axons}\in \left[-1,1\right]$$

The statistical significance of the changes in proximal-to-distal poly(A) site ratio between cell body and axons was assessed by Fisher’s Exact Count Test using summed-up raw read counts of promoter-proximal versus promoter-distal 3′ UTR isoforms originating in the cell body or axonal samples. We applied a False Discovery Rate adjusted threshold of 0.01. A shift toward the usage of promoter-proximal isoforms in axons compared to cell body was considered when $S_{1}\leq -1$, $S_{2}\leq -15\text{\%}$ and FDR < 0.01. A shift toward the usage of promoter-distal isoforms in axons compared to cell body was considered when $S_{1}\geq 1$, $S_{2}\geq 15\text{\%}\;$and FDR < 0.01. Finally, a stringent threshold was applied to identify highly enriched isoforms in axons as following: for those tandem 3′ UTR isoforms showing shift toward the usage of promoter-proximal isoforms in axons as compared to cell body, we required ${\frac{I_{proximal}}{I_{proximal}+I_{distal}}}_{CB}\leq 0.2$. Conversely for those tandem 3′ UTR isoforms showing shift toward the usage of promoter-distal isoforms in axons, we required ${\frac{I_{proximal}}{I_{proximal}+I_{distal}}}_{CB}\geq 0.8.$

#### Gene Ontology (GO) enrichment analysis

GO analysis was performed by comparing pairs of gene lists using the Fisher Test with the topGO Bioconductor package https://bioconductor.org/packages/release/bioc/html/topGO.html. Only GO terms containing at least 10 annotated genes were considered. We applied a P value threshold of 0.05. We manually filtered biologically relevant and statistically enriched GO by removing redundant GO terms and those applying to fewer than 5 genes in the gene lists.

#### RT-PCR and quantitative RT-PCR

mRNA was isolated from sympathetic neurons or PC12 cells using TRIzol or RNAeasy mini Kit (QIAGEN) and reverse transcribed with random hexamers and SuperScript III or IV. RT-qPCR reactions (20 μL) contained 10 μL of Flash SybrGreen Mastermix, or 12.5 μL of SybrSelect Mastermix and 0.25 μM primers, unless otherwise indicated. Reactions were performed in duplicate or triplicate with the Mastercycler® Realplex (Eppendorf) or Biorad CFX qPCR machines. For absolute quantification, each experiment included a standard curve, a no-RT control and a no-template control. Standard templates consisted of gel-purified PCR amplicons of known concentrations and each standard curve consisted of seven serial dilutions of the DNA template. For relative quantification, the Comparative Ct Method (ΔΔCt Method) was used. At the end of 40 cycles of amplification, a dissociation curve was performed in which SybrGreen fluorescence was measured at 1°C intervals between the annealing temperature and 100°C. Melting temperatures of amplicons varied between 80°C and 92°C. Primer sequences and PCR conditions are described in Table S4.

#### Northern blotting

RNA purified from SCG neurons cultured for 7 days or from PC12 cells was separated by electrophoresis in denaturing conditions and transferred to nylon membrane by capillary blotting according to standard protocols. Probes corresponding to IMPA1, Firefly Luciferase or GAPDH coding sequences, or IMPA1-L 120nt fragment were labeled using Random Priming Labeling Kits (Takara or Roche) and [α-^32^P] dCTP. Blots were exposed to films or phosphorimager screens and radioactive signal was quantified using ImageJ or ImageQuant TL software (GE Healthcare), respectively.

#### Tag Addition-PolyAdenylation Test (TA-PAT)

Poly(A) tail length test was performed using the USB Poly(A) tail length assay kit following the manufacturer’s instructions. Briefly, total RNA was purified from PC12 cells and tagged by G/I tailing to the end of the mRNA using PolyA polymerase (37°C, 60min). The tagged RNA was reverse transcribed (44°C, 60min) using the kit reverse transcriptase and a primer that anneals to the G/I tail. cDNA was then amplified using the IMPA1-1276 or IMPA1-2027 Forward primers that anneal just upstream of the IMPA1-C or IMPA1-L cleavage site, respectively. For the isoforms generated by Firefly constructs carrying IMPA1-L or IMPA1-L Δ PAS 3′ UTR, the TA-PAT assay was performed with a nested PCR format to exclude amplification from endogenous IMPA1 transcripts isoforms. Primer sequences and PCR conditions are described in Table S4.

#### 3′ Rapid Amplification of cDNA Ends (3′ RACE)

Full length 3′ UTR of *IMPA1*, *actin beta*, *stathmin 2* and *cofilin1* mRNAs were amplified from axonal and cell body compartments by performing 3′ RACE reactions on total RNA isolated from compartmentalized chambers as previously described (Andreassi et al., 2010). Samples were concentrated by speed-vacuum, RNA was divided in two equal samples and used for amplification with SMART RACE cDNA Amplification Kit (Clontech) according to manufacturer’s instructions. Gene specific primers for 3′ RACE assays are listed in Table S4. Amplification was performed using Advantage GC 2 PCR kit (Clontech) and PCR products were cloned and sequenced.

#### Cloning

IMPA1 Cleaved (IMPA1-C) and Long (IMPA1-L) 3′ UTR sequences were amplified by PCR from the corresponding RACE clones. After digestion with *NotI*/*XhoI*, IMPA1-C DNA fragment was purified and used to replace IMPA1-L in myrdEGFP-IMPA1-L (Andreassi et al., 2010). Mouse IMPA1 coding sequence was PCR-amplified from mouse brain cDNA using primers encoding the HA tag. After digestion with BamHI/*NotI*, HA-*ms* IMPA1 DNA fragments were purified and used to replace myrdEGFP sequence in myrdEGFP-IMPA1-L or myrdEGFP-IMPA1-C. The 120 nts localization signal of rat IMPA1-L was cloned by PCR from an IMPA1-L RACE clone and cloned at the 3′ of HA- ms IMPA1-C plasmid to generate IMPA1-C+120 3′ UTRs. To generate firefly reporter vectors, Firefly luciferase coding sequence was PCR amplified from pGL3 vector (Promega) with primers containing restriction sites for BamHI and *NotI*. The DNA fragment was purified and used to replace the myrdEGFP sequence in myrdEGFP-IMPA1-C or myrdEGFP-IMPA1-L. Mutation of IMPA1-C and IMPA1-S poly(A) sites (PAS), and IMPA1 Cleavage site was performed by PCR site-directed mutagenesis (Agilent) of Firefly-IMPA1-L vector. In all Firefly constructs, the bovine Growth Hormone polyadenylation signal was removed by PCR site-directed mutagenesis by creating an extra *XhoI* site, that was used for digestion and re-ligation.

Full-length mouse Ago2 cDNA in pCMV6 entry vector was purchased from Origene and verified by sequencing. Catalytic mutant (CD) version of the protein was generated by mutation of aspartate 597 to alanine (Liu et al., 2004) using site-directed mutagenesis PCR (QuikChange Lightning Kit, Agilent) as per manufacturer’s instruction. To clone Ago2 in pGEX 4T-2 bacterial expression vector (GE Healthcare Life Sciences), pCMV6 vectors carrying wild-type or mutant Ago2DNAs were digested with BamHI, filled-in with Klenow polymerase (NEB), digested with *NotI* and band isolated. The pGEX 4T-2 vector was digested with SmaI and *NotI*, purified and then ligated to the filled-in/*NotI* wild-type or CD Ago2 sequences. Cloning was confirmed by sequencing. Primer sequences and PCR conditions are described in Table S4.

#### Electroporation and analysis of mRNA transport in axons

Neurons were electroporated with the indicated constructs as previously described (https://www.cellectricon.se/pdf/Sympathetic_neurons.pdf). MyrdEGFP was detected by GFP immunostaining. Confocal images were acquired with a SP5 confocal system (Leica) using LAS AF software and automated tiling over several z stacks, to cover the whole thickness and length of the axons. Maximal intensity projections were processed with Fiji software. Axons were traced manually using NeuronJ plugin and gray value intensity over length was measured. Data analysis was performed using Excel software to calculate average values and standard error means of the intensity for each 200 μm axonal segment.

#### smFISH and immunofluorescence

Probe sets targeting the 3′ UTR of rat *Nefl*, *Snrk*, *Abpa2* and *IMPA1-L* (Stellaris probes, Biosearch Technologies) were designed using the Stellaris probe-set designer tool to specifically detect the long 3′ UTR isoforms of the transcripts. *Nefl*, *Snrk* and *Abpa2* probes were 3′end labeled with CalFluor590. *IMPA1-L* probes were 3′end labeled with Quasar570. Probes were reconstituted at 12.5 μM in TE buffer (10mM Tris pH8, 1mM EDTA pH8). smFISH was performed as previously described (Crerar et al., 2019). SCG neurons were cultured 4-7 days *in vitro* on glass coverslips, washed with PBS and fixed using 3.7% PFA at RT for 10mins. Cells were permeabilized with 70% EtOH at 4°C for 3hrs and then pre-hybridized in 2xSSC 10% Formamide for 5mins at RT. 1 μL of 12.5 μL probe stock was added to 100 μL Hybridization buffer (10% Dextran Sulfate, 2xSSC, 10% Formamide, 200mM vanadyl ribonucleoside, 0.02% RNase-free BSA, 0.1mg/mL tRNA) and coverslips were incubated O/N at 37°C in humidified chamber. Coverslips were then washed 2x in warm 2xSSC 10%Formamide at 37°C for 30mins in the dark. For smFISH+IMF, coverslips were then blocked in 1%BSA 2xSSC for 30mins RT before incubation with anti-Ago2 or Anti-HuD antibodies in 1%BSA 2xSSC O/N at 4°C. Coverslips were subsequently washed 3x5mins RT in 2xSSC before incubation with secondary antibodies +100ug/mL DAPI in 2xSSC 45mins 37°C, followed by 3x5mins RT in 2xSSC washes. Coverslips were mounted to slides with ProlongGold, cured O/N at RT before imaging on a 3i confocal microscope (Intelligent Imaging Innovations, Inc.) equipped with a Photometrics Prime 95B (Scientific CMOS) camera. For immunofluorescence only staining, the same protocol was performed except permeabilization was done using 0.3% Triton X-100 in PBS and blocking in 15% normal goat serum. All washes and solutions were in PBS. Images were then processed using ImageJ software. Antibodies and probing conditions are described in Table S4.

#### Quantification of axon degeneration

SCG explants were grown for 36 hours before electroporation with the indicated siRNAs (150nM, GE Dharmacon) and a GFP expression vector (20ng/μL), in the presence of either HA-*ms* IMPA1- C, HA-*ms* IMPA1- C+120, or HA-*ms* IMPA1- L DNAs, (200 ng/μL), as indicated. After 6 days, GFP fluorescence was detected with an inverted Leica epifluorescence microscope, and intact axon bundles that showed no sign of breakdown (i.e., the classical beads-on-string morphology of degenerating axons) were quantified. For imaging, explants were fixed in 4% PFA and stained with anti-GFP and anti-neurofilament antibodies. Antibodies and probing conditions are described in Table S4.

#### Co-immunoprecipitation and western blotting

Co-immunoprecipitation samples were obtained by lysing cells in RIPA buffer (50mM Tris-HCl pH 7.4, 150mM NaCl, 1% NP-40, 0.5% Sodium deoxycholate, 0.1% SDS, 1mM EDTA, Protease Inhibitors Cocktail) for 10min on ice. After centrifugation, protein concentration in the supernatants was assayed by Pierce™ BCA Assay, and 0.5-1 mg of pre-cleared protein sample was incubated with 2 μg of antibody as indicated, overnight at 4°C, on constant rotation. Immuno-complexes were precipitated by adding protein A-agarose beads (GE Healthcare) at 4°C for 2hrs. After extensive washes with RIPA buffer, immune-complexes were eluted from the beads by boiling in 1X LDS-buffer +2.5% βmercaptoethanol. Samples were resolved on 4%–12% PAA pre-cast gels and blotted on PVDF membrane (Amersham). For western blotting, cells were rinsed with PBS and lysed in the plates with 1X LDS-buffer +10% βmercaptoethanol. SDS-PAGE and blotting was then performed as described above. For immunodetection, membranes were blocked in 5% milk for 1hr at room temperature and incubated overnight with the indicated antibodies. Antibodies and probing conditions are shown in Table S4.

#### RNA ImmunoPrecipitation (RIP)

RNA immunoprecipitation was performed as described (Napoli et al., 2008) with minor modifications. Briefly, protein A/G agarose beads (Santa Cruz) were incubated with antibody (5 μg in 1% BSA in PBS) and heparin (1mg/ml) for 2hrs at 4°C, washed with washing buffer (150mM NaCl, 50mM Tris-HCl [pH 8.0], 1% Triton X-100), and incubated with 250-300 μg of protein lysates 1 hr at 4°C. Beads were extensively washed, and RNA was eluted in 0.2M Na Acetate, 1mM EDTA, and 0.2% SDS for 5 min at 70°C. For normalization, 20pg of *in vitro* transcribed RNA synthesized from the T7 control DNA Template (AmpliScribe T7 Transcription Kit, Epicenter) was added to the samples. RNA from inputs and immunocomplexes was purified, subjected to DNase digestion (Ambion), reverse transcribed and assayed by qPCR. Primer sequences and PCR conditions are described in Table S4.

#### Dual luciferase assay

PC12 cells were transfected with the indicated Firefly Luciferase-IMPA1 constructs and thymidine-kinase promoter– Renilla Luciferase (Promega) using Lipofectamine 2000 and incubated for 48hrs. Samples were processed using the dual-luciferase reporter assay system (Promega), according to manufacturer’s instructions.

#### Polysome fractionation

Polysome fractionation was performed as described (Johannes and Sarnow, 1998). Briefly, PC12 cells were lysed in ice-cold gradient buffer (0.3M NaCl, 1mM MgCl2, 15mM Tris-HCl (pH7.4), 0.1mg/mL cyclohexamide and 1mg/mL heparin, 1% Triton X-100, 500U/mL RNase inhibitors). Samples were centrifuged and the supernatants layered onto 10%–50% sucrose linear gradients. The gradients were sedimented at 38,000 rpm, using a SW40Ti rotor (Beckman) or a Sorvall TH-641 rotor for 2 hr at 4°C. Eleven fractions (1mL each) were collected from the gradients and transferred in 3ml of 7.7M guanidine-HCL using a Foxy R1 gradient fractionator (Teledyne ISCO; ISCO peak Trak version 1.10 software) with continuous measurement of the absorbance at 254nm. RNA was precipitated, treated with DNase and purified using RNAeasy Mini Kit (QIAGEN). For fractions 1 and 2, protocol was modified as suggested by manufacturer for recovery of small size RNA. Samples were concentrated by speed-vacuum and analyzed by Northern blot.

#### Mass spectrometry

Immuno-complexes were precipitated from 20x10^6^ PC12 cells naive or differentiated with NGF for 4 days. On-bead digestion and nano LC-MS/MS analysis was performed as described (Bernaudo et al., 2015) with minor changes. The procedure is summarized in Figure S6 and described briefly below. Immuno-precipitated proteins were released from the resin by on-beads digestion for 15min at 37°C using 200ng of trypsin (Promega). The supernatants were collected and subjected to conventional in-solution tryptic digestion (overnight at 37°C) in denaturing conditions (reduction by 10mM DTT for 1hr at 37°C followed by 24mM iodoacetamide for 1hr at 37°C quenched by addition of 2mM DTT for 30min at 37°C). Tryptic peptides were then subjected to differential labeling by oxygen18 (Boersema et al., 2009). Pairs of differently labeled samples were mixed, purified by StageTips (Rappsilber et al., 2007) and subjected to nano LC-MS/MS analysis. Chromatography was performed on an Easy LC 1000 nanoLC system (Thermo Fisher Scientific, Odense, Denmark). The analytical nanoLC column was a pulled fused silica capillary, 75 μm i.d., in-house packed to a length of 10 cm with 3 μm C18 silica particles from Dr. Maisch GmbH (Entringen, Germany). A 60-min binary gradient was used for peptide elution. MS detection was performed on a quadruple-orbitrap mass spectrometer Q-Exactive (Thermo Fisher Scientific) operating in positive ion mode and data-dependent (Top-12) scanning mode. Data were processed using Proteome Discoverer 1.4 (Thermo Fisher Scientific), using Sequest as search engine, and querying the March 2015 RATTUS reference proteome sequence database (UniProt: http://www.ebi.ac.uk/uniprot). The protein sequence database was merged with a list of common contaminants named “Common Repository of Adventitious Proteins” retrieved from The Global Proteome Machine website (https://www.thegpm.org/crap/index.html). In total, 27,927 entries were searched. Peptide identifications were validated by Percolator (Käll et al., 2007) integrated in Proteome Discoverer. Percolator q-value was set to equal or less than 0.05. Quantification values based on < 3 peptides were manually checked in raw MS data. MS/MS data relative to protein hits identified by a single peptide are reported in Table S3. Protein H:L ratios obtained from all technical replicates of a given biological replicate were transformed into log2 space before their median was calculated.

#### RNA oligonucleotide-Mediated Ligation (RML) RT-PCR and cloning

RML RT-PCR was performed as described (Endres et al., 2011) with the following modifications. Cleaved fragments were isolated and cloned using 1.2ng of axonal RNA purified and pooled from 55 explants where the cell bodies had been surgically removed, or 1.5 μg or less of total cellular RNA. Total cellular RNA was DNase-digested and purified by phenol:chloroform purification. Quality control of starting material was performed using Agilent Tapestation 2200 (UCL Genomics). Samples with a RIN value ≥ 7.2 were used for RLM RT-PCR. RNA was denatured and tagged by ligation with 25ng or 250ng of RNA oligo for axonal or total RNA, respectively, and 30U of T4 RNA ligase (NEB) for 1hr at 37°C followed by overnight incubation at 16°C in a PCR machine. Ligated axonal RNA was then purified using buffer PB (QIAGEN) +10% β-mercaptoethanol and AMPure XP beads (Beckman Coulter) as per manufacturer’s instructions. Purified ligated RNA was reverse transcribed using random hexamers and 50U SuperScript IV reverse transcriptase for 1hr at 50°C. After RNaseH (NEB) digestion, cleaved fragments were amplified by PCR using Q5 DNA polymerase (NEB) and cloned in pCR4Blunt-TOPO® vector according to manufacturer’s instruction. For restriction digestion analysis, it is important to note that EcoRI sites flank the PCR product insertion site for excision of the insert. At least 7 individual, random clones were analyzed by sequencing. When used for RT-qPCR, amplification of the cleaved fragments was carried out in 25 μL reaction using SybrSelect MasterMix. Primer sequences and PCR conditions are described in Table S4.

#### Production of recombinant Ago2 proteins

Full-length mouse wild-type or catalytic mutant (CD) Ago2 were purified as GST-fusion proteins from *E. Coli* BL21 Star OneShot (ThermoFisher Scientific) transformed with ms Ago2 WT or CD in pGEX-4T 2 plasmids. Cell cultures were induced for 18hrs at 22°C with 10mM isopropyl β-d-thiogalactopyranoside (IPTG) and then collected by centrifugation at 6000rpm for 6 minutes in a Beckman JLA-10.500 rotor. The cell pellet was resuspended in 20 mL of PBS supplemented with 10mM DTT, 5mM Mg(OAC)_2_ and 1:100 protease inhibitor mixture (PIC, Sigma) and lysed by sonication (Branson sonicator). The lysate was cleared by spinning at 11000rpm for 22 minutes in a Beckman JA-25.50 rotor. Recombinant proteins were purified using GSTrap FF Columns (GE 17513001) and a GE Akta Purifier 10 chromatographing system following the manufacturer’s instructions. The eluates were concentrated using Amicon filter units Ultra-0.5 (30KDa cut off, Sigma) as per manufacturer’s instructions. The purified proteins were then analyzed by Coomassie staining of SDS-PAGE gels and yield estimated by comparison to known amounts of purified BSA protein. The purified proteins were then stored at −80°C.

#### Radioactive *in vitro* cleavage assay

Assays were performed as described with the following modifications (Miyoshi et al., 2008). RNA oligos were prepared by *in vitro* transcription using mirVana probe construction kit according to manufacturer’s instruction. RNA was then dephosphorylated using Calf Intestine Phosphatase (NEB) and purified by phenol:chloroform extraction. After precipitation, RNA probes were labeled at the 5′ using [γ-^32^P]-ATP and T4 polynucleotide kinase (ThemoFisher Scientific). After gel purification of full-size probes, oligos were incubated for 2 hr at 26°C with cytoplasmic protein fractions prepared from sympathetic neurons using NE-PER kit (Pierce). *In vitro* cleavage assays were performed by adding 50nM human recombinant Ago2 (expressed in Baculovirus, Active Motif) or home-made mouse wild-type (WT) or catalytic mutant (CD) Ago2 in a reaction mixture containing 25mM HEPES-KOH pH 7.5, 50mM KOAc, 5mM Mg(OAc)_2_, 5mM DTT for 1.5 hr at 26°C. When testing Ago2 biological activity, 30nM recombinant Ago2 was incubated for 2 hr at 26°C with 30nM single-stranded, phosphorylated *luc* or *IMPA1* guide siRNA prior to cleavage assay of *Luc* or *IMPA1* RNA target RNA oligos. Following purification using TRizol, samples were separated on 8% acrylamide gel in denaturing conditions and gels were exposed to X-rays. Oligos sequences are listed in Table S4.

### Quantification and statistical analysis

Data are expressed as means ± SEM. Unless otherwise noted in the Figure legend, one-way ANOVA with post hoc test or t test were used as indicated to test for statistical significance, which was placed at p < 0.05. In all experiments, each data point refers to independent biological replicates from independent cell cultures and experiments. For all data except RNA-seq data, statistical analyses were performed using GraphPad Prism software 7.01.

## Supplemental information

Document S1. Figures S1–S7 and Tables S1–S5

Document S2. Article plus supplemental information
