# Supplementary material for: Cytoplasmic cleavage of IMPA1 3′ UTR is necessary for maintaining axon integrity
Source: Cell Rep. 2021 Feb 23;34(8):108778. doi: 10.1016/j.celrep.2021.108778 (PMC7918530; doi:10.1016/j.celrep.2021.108778)
Supplement: Document S1. Figures S1–S7 and Tables S1–S5 [file mmc1.pdf]

Cell Reports, Volume *34*

Supplemental information

Cytoplasmic cleavage of *IMPA1* 3′ UTR

is necessary for maintaining axon integrity

Catia Andreassi, Raphaëlle Luisier, Hamish Crerar, Marousa Darsinou, Sasja Blokzijl-Franke, Tchern Lenn, Nicholas M. Luscombe, Giovanni Cuda, Marco Gaspari, Adolfo Saiardi, and Antonella Riccio

Fig. S1

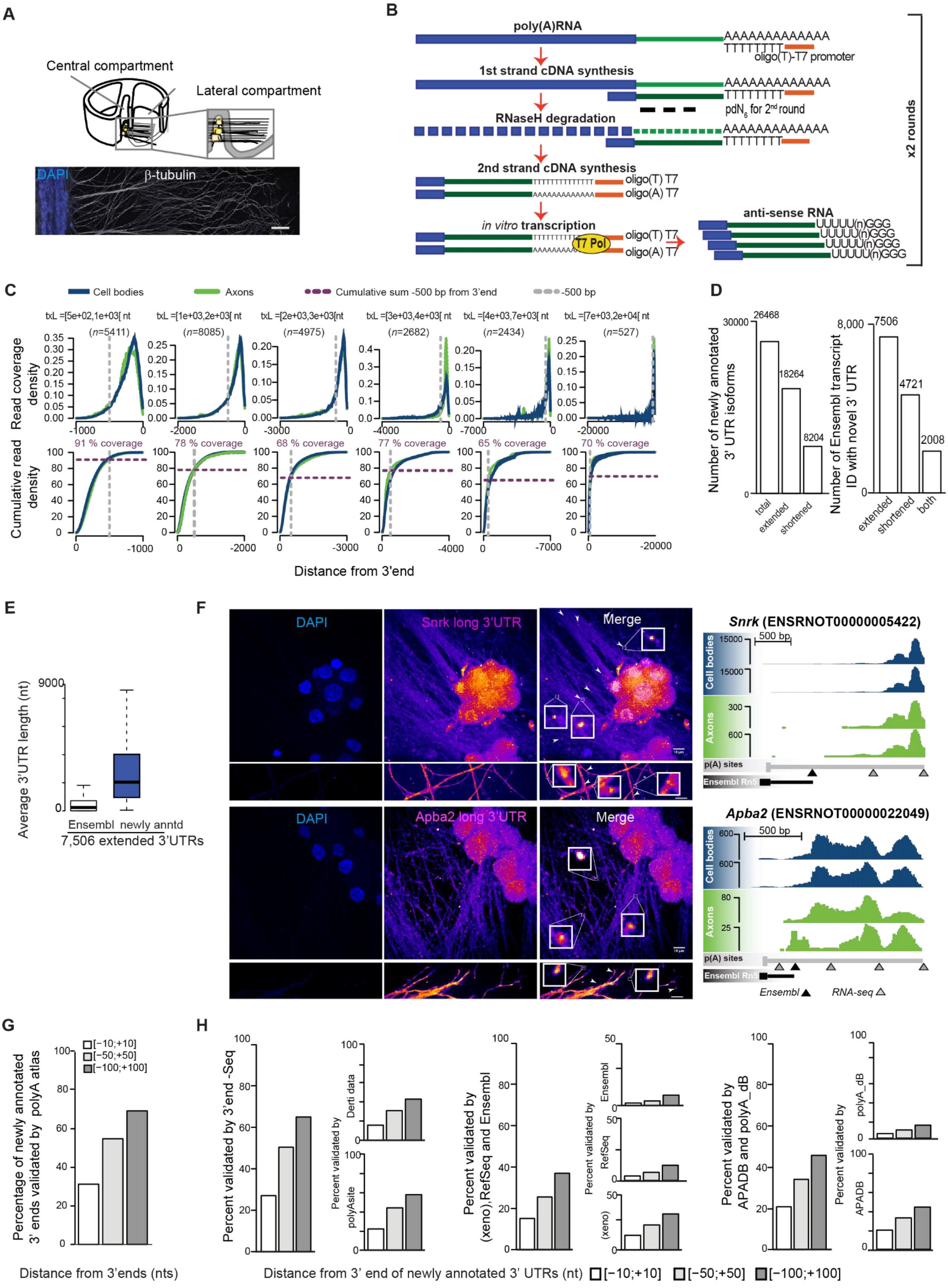

Figure S1. 3' end-RNA-seq of transcripts in axons and cell bodies of sympathetic neurons.

Related to Figure 1.

(**A**) (*Upper panel*) Schematic representation of a compartmentalized chamber. (*Lower panel*) Staining of sympathetic neurons grown in compartmentalized chambers for 10 days with DAPI and anti-α tubulin antibody. Scale bar=500µm.

(**B**) Workflow of linear amplification of mRNA.

(**C**) Reads accumulation at 3’ end of the Ensembl transcripts in function of transcript length. (*Upper*) Read density coverage and (*Lower*) cumulative read density along transcript are shown.

(**D**) (*Left*) Number of newly annotated 3’UTR isoforms compared with Ensembl Rn5 annotations. (*Right*) Number of Ensembl transcript ID expanded with newly annotated 3’UTRs.

(**E**) Average length of the 3’UTRs of 7,506 Ensembl transcript ID extended by intersecting expressed genomic fragments with Ensembl Rn5 annotation.

(**F**) (*Left*) smFISH of *Snrk* and *Apba2* long 3’UTR in sympathetic neurons cell bodies and axons. Arrowheads indicate mRNA puncta without any pixel dilation. Insets= 5X magnification of boxed area (scale bar=10μm). (*Right*) Genome browser view of *Snrk* and *Apba2* in axons and cell bodies.

(**G**) Percentage of newly annotated 3’ ends recovered from a polyadenylation site atlas (see STAR Methods) at the indicated distance intervals from novel 3’ ends.

(**H**) Percentage of 3’UTR isoforms for which the indicated region surrounding the 3’ end intersects with a PAS obtained from 3’ end RNA-seq data, a 3’ terminus annotated in RefSeq (Rn5, Rn6 and XenoRefSeq) or Ensembl (Rn6), or a PAS annotated in APADB or PolyA_DB2. Comparison between combined (*Left*) and individual (*Right*) datasets is shown in each panel.

Fig. S2

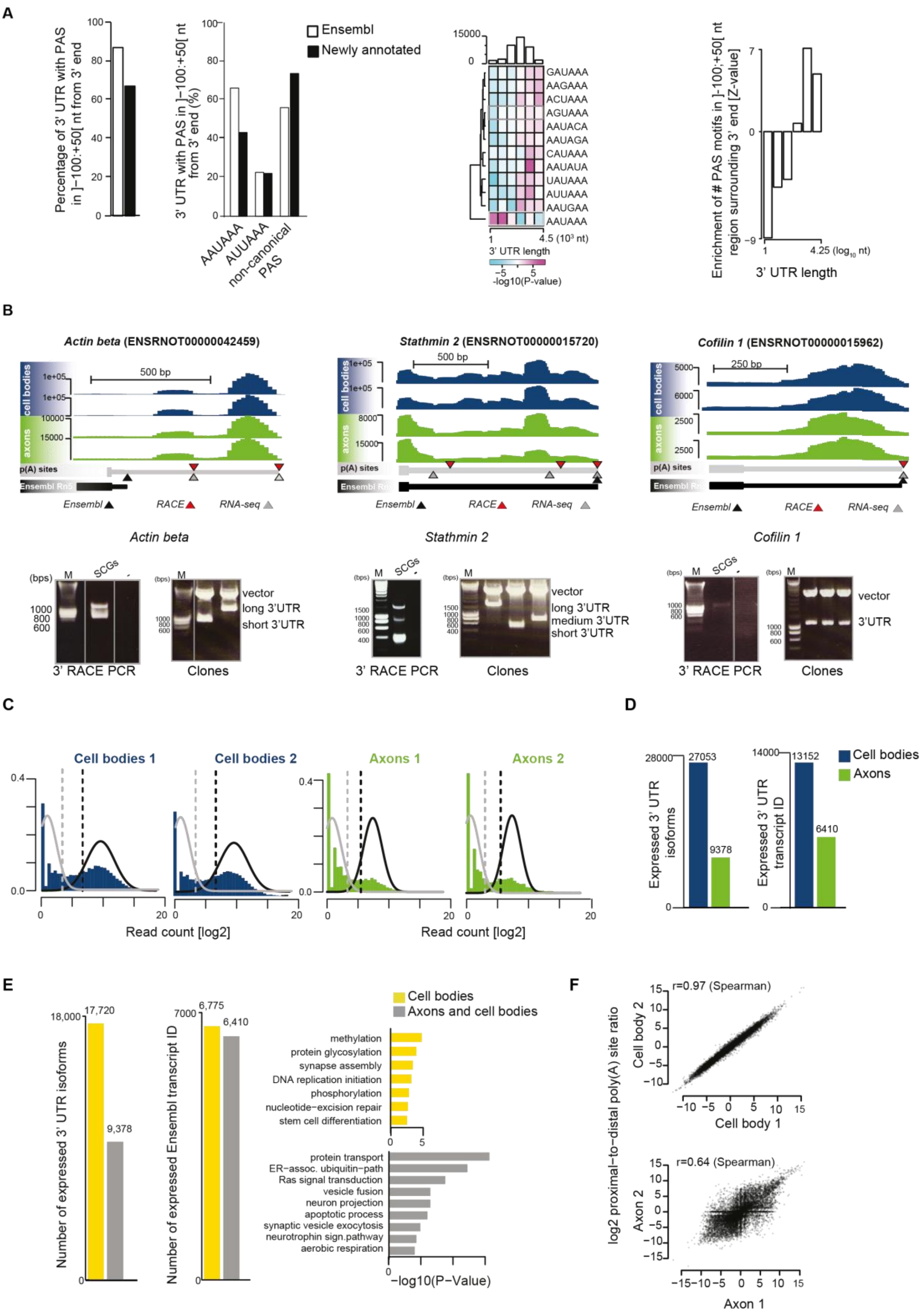

Figure S2. Analysis of 3'UTR PAS choice in axons and cell bodies of sympathetic neurons. Related to Figure 2.

(**A**) (*Left*) Frequency of canonical and variant PAS motifs detected between -100 to +50 nt of

newly annotated (black) or Ensembl annotated (white) 3' ends. Total PAS motifs and canonical *vs.* variant PAS motifs are shown. (*Middle*) Relative occurrence of different PAS motifs in promoter-proximal and promoter-distal 3'UTRs. Upper column graph indicates the number of 3'UTR isoforms per range of 3'UTR length. Color-scale: -$\log_{10}$(P- value) of enrichment in PAS motif obtained by Fisher test of the number of 3' ends that contains at least one motif per range of 3'UTR length. (*Right*) Relative occurrence of PAS motifs in the [-100;+50] nt region surrounding the 3' ends at increasing length.

(**B**) (*Top*) Genome browser view of the *Actin beta*, *Stathmin 2* and *Cofilin 1* 3'UTRs. 3' end isoforms annotated in Ensembl Rn5 or identified by RNA-seq data and by RACE are indicated by arrowheads. (*Bottom*) Agarose gel analysis of RACE PCR products to amplify *Actin beta*, *Stathmin 2* and *Cofilin 1* 3'UTRs (*Left*) and of restriction digestions of representative clones obtained by cloning of corresponding RACE PCR products (*Right*). Noncontiguous lanes from the same experiment and gels are shown side by side, as indicated by lines in the figure.

(**C**) Identification of 3'UTR isoforms expressed in cell bodies (blue) and axons (green) performed by fitting bimodal distribution on $\log_2$-raw count mapping the 500 nts distal region of 3' end.

(**D**) Number of 3'UTR isoforms (*Left*) and Ensembl transcript ID (*Right*) expressed in cell bodies and axons.

(**E**) Comparative analysis of 3'UTR isoforms and transcript IDs enriched in cell bodies (yellow) and axons (grey) (L*eft*). Statistically enriched GO terms of genes identified in cell bodies and axons samples *(Right*).

(**F**) Scatter plots of the relative use of promoter-proximal and promoter-distal poly(A) sites in two biological replicates of cell body (*Upper*) and axon (*Lower*) samples.

Fig. S3

A

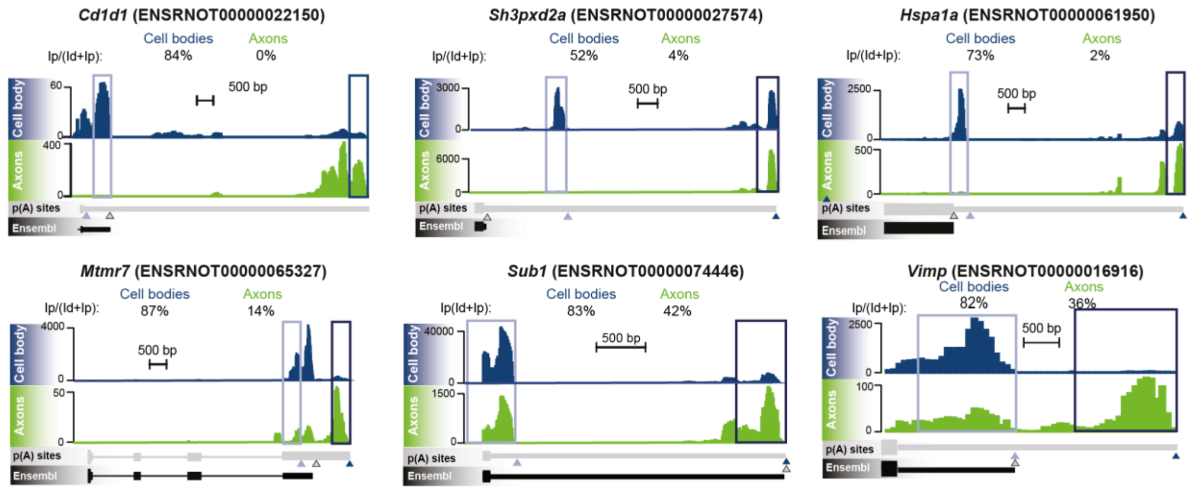

B

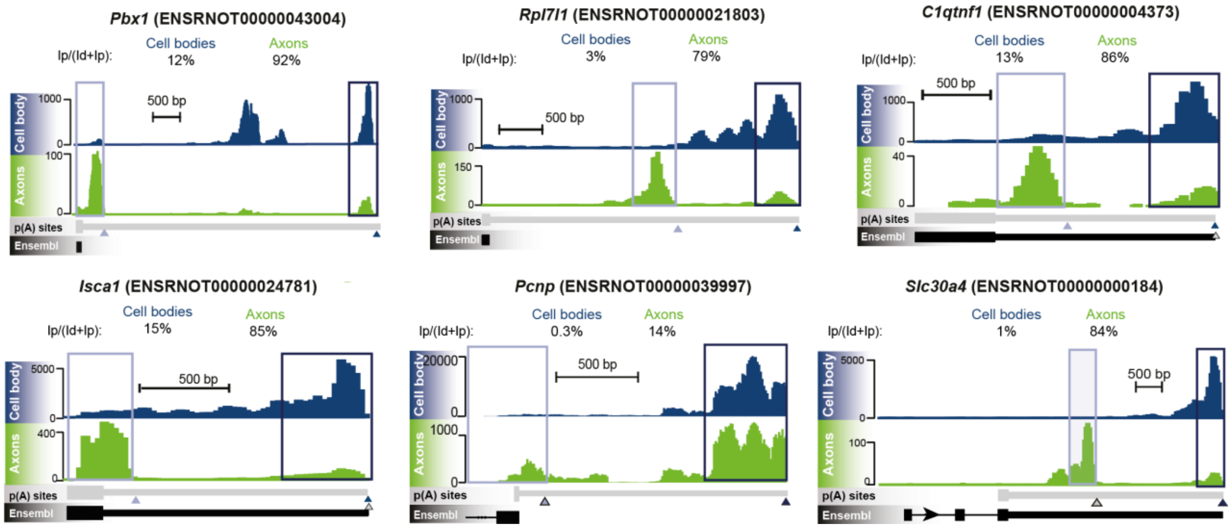

Figure S3. Axonal transcripts with a proximal or distal 3'UTR bias. Related to Figure 3.

(**A** and **B**) Examples of transcripts with a marked shift towards (**A**) increased promoter-distal poly(A) site usage or (**B**) increased promoter-proximal poly(A) site usage, in axons compared to cell bodies.

Fig. S4

A

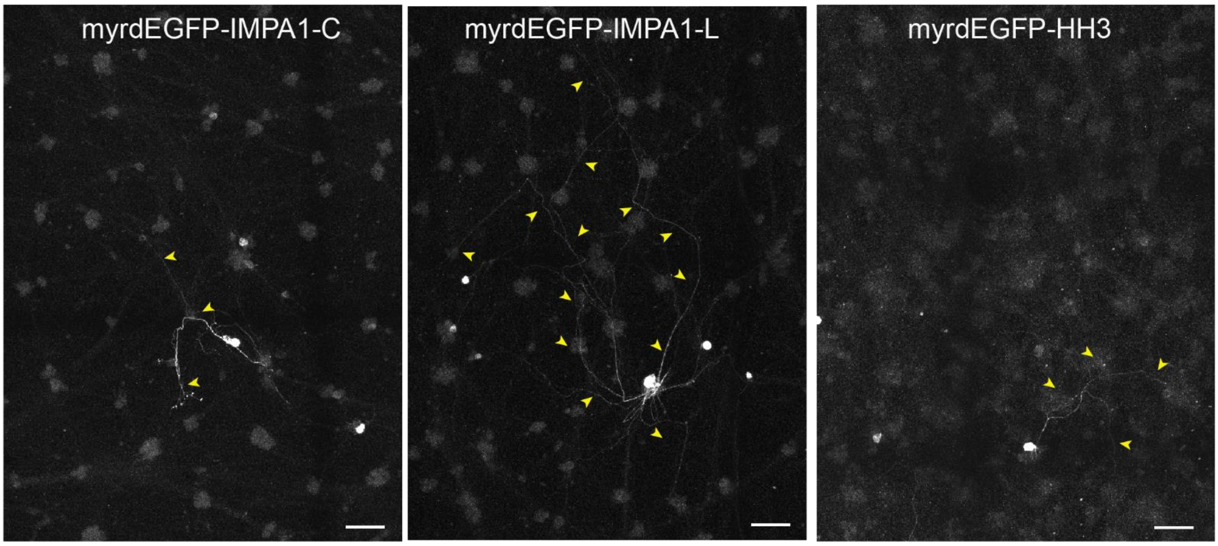

B

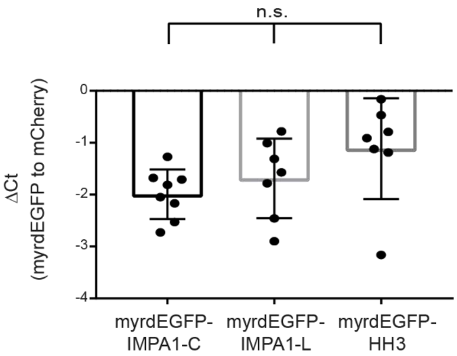

C

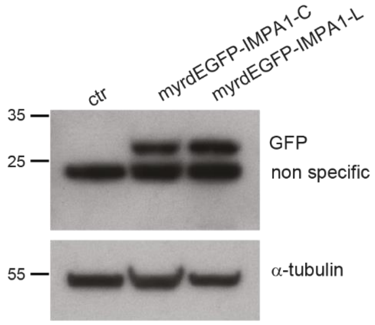

D

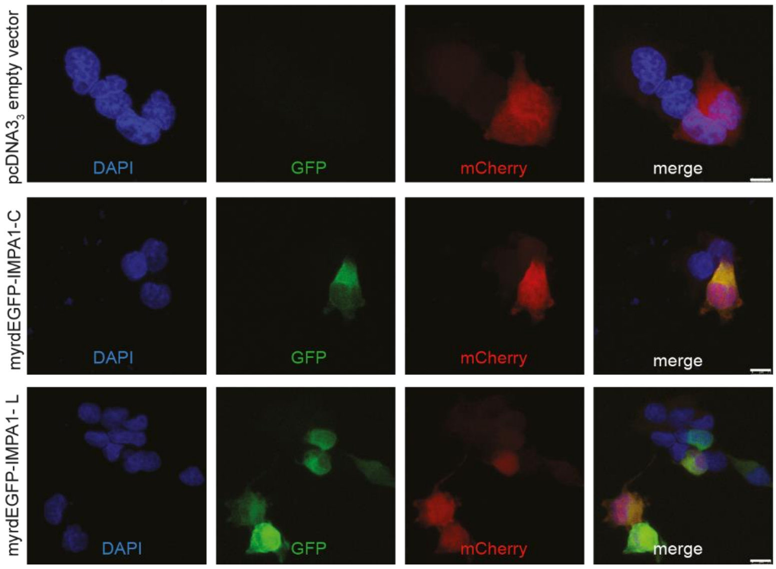

Figure S4. A dEGFP reporter system to study IMPA1 3'UTR localization. Related to Figure 4.

(**A**) GFP-immunostaining of sympathetic neurons transfected with either *myrdEGFP-IMPA1-C*, *myrdEGFP-IMPA1-L* or *myrdEGFP-Histone H3 (HH3).* Arrowheads indicate GFP immunoreactivity along the axons of transfected neurons. Scale bar=200μm.

(**B**) RT-qPCR of *myrdEGFP-IMPA1-C*, *myrdEGFP-IMPA1-L* or *myrdEGFP-Histone H3* (HH3) transfected in cell bodies excised from sympathetic ganglia explants and normalized by a co-transfected vector expressing mCherry. Data are mean± s.e.m. of ΔCt of myrdEGFP Ct normalized to mCherry Ct. One way Anova, non-statistically significative (n≥7).

(**C**) Western blot analysis of GFP and α-tubulin on PC12 cells transfected with either myrdEGFP-IMPA1-C or myrdEGFP-IMPA1-L. Irrelevant lanes have been excluded. Ctr: non transfected cells.

(**D**) DAPI, anti-GFP and anti-mCherry staining of naïve PC12 cells co-transfected with empty vector or myrdEGFP-IMPA1-C or myrdEGFP-IMPA1-L, and mCherry plasmids. The non-specific band detected by western blotting does not affect the immunofluorescence staining. Scale bar=75μm. (n=3).

Fig. S5

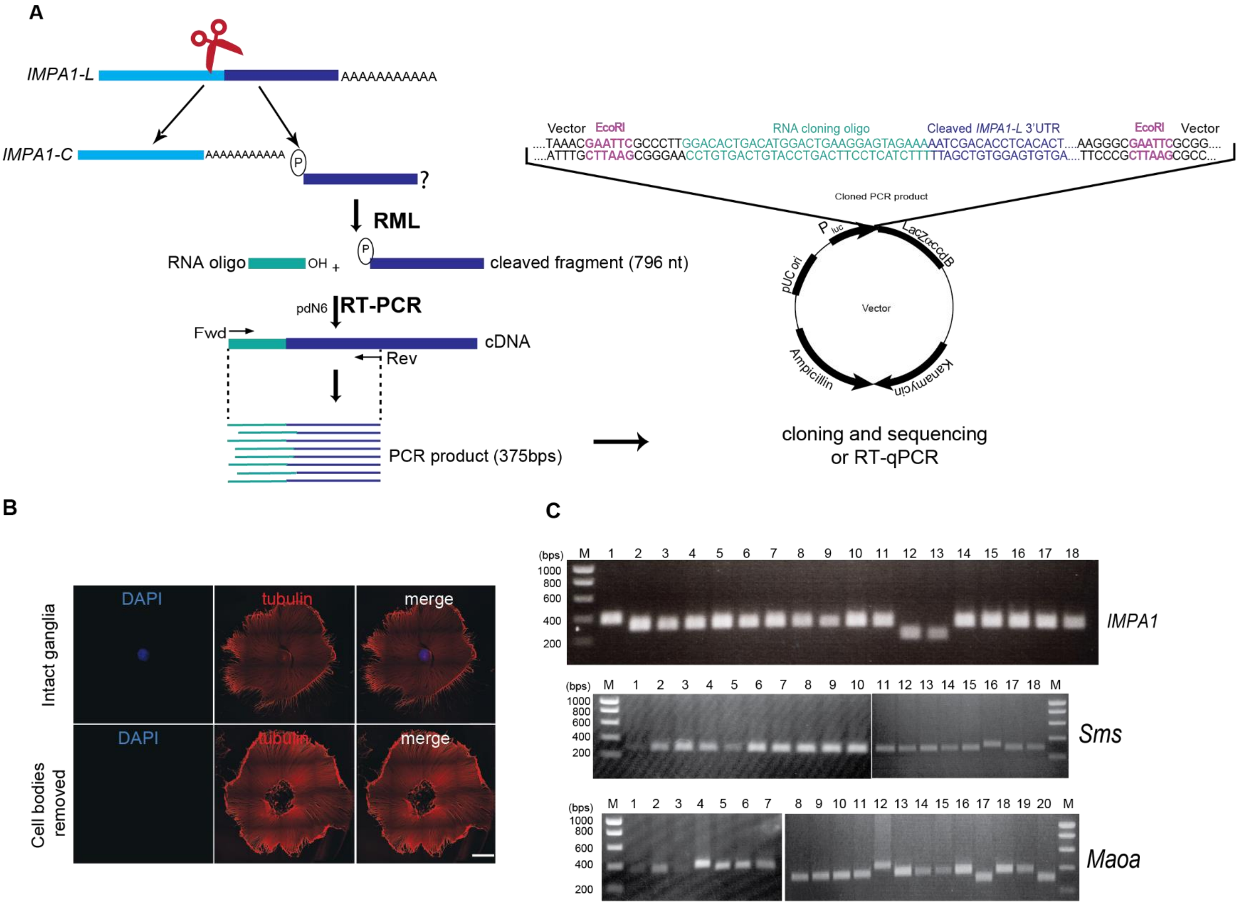

D

Gene	Chromosome	Proximal PAS position	Fragment starting position	Sequence at 5' end of fragment	Number of RML clones carrying this sequence	Distal PAS position
Impa1	Chr2 Fwd strand	93695533	93695572	5'-AATCGACACCTCACACTGAGTGCTTGAAGCTGCAATTCCTGCTTC..	15 out of 17	93696336
Sms	Chr10 Fwd strand	40415097	40415125	5'-GTCCCTTTATTTCAGCCCCTGTTCCTCTTTCTTAACTGCTGATGGC...	19 out of 19	40727788
Maoa	ChrX Rev strand	6555153	6555141	5'-TACAGCCTATGGTTTGGGCCATTTAAGTGCACTTGATTTAACCC.......	17 out of 19	6553338

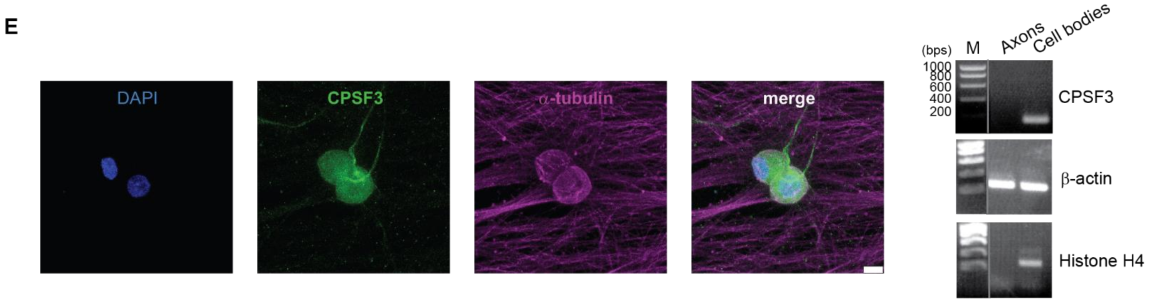

Figure S5. Analysis of axonal cleavage of transcripts by RML-RT-PCR. Related to Figure 5.

(**A**) Schematic representation of 5'P-dependent RNA oligo-Mediated Ligation (RML) and

cloning experiments.

(**B**) DAPI and anti-α tubulin staining of SCG explants before (*Top*) or after (*Bottom*) the removal of cell bodies. Scale bar =200μm.

(**C**) *EcoRI* restriction digestions of clones carrying an insert corresponding to the cleaved fragments of IMPA1-L (*Left*), Sms (*Right*) and Maoa (*Bottom*) in axons. Noncontiguous lanes of 2 agarose gels from the same experiment are shown side by side, as indicated by the grey line.

(**D**) Genomic coordinates (Ensembl Rnor_6.0) for the proximal and distal PAS, and for the 5'ends of the cleaved fragments of IMPA1, Sms and Maoa are listed, together with the sequence at the 5' end of the cleaved fragments as obtained by sequencing of the indicated number of clones.

(**E**) (*Left*) DAPI, anti-CPSF3 and anti-α tubulin staining in axons and cell bodies of sympathetic neurons. Scale bar=10μm. (*Right*) RT-PCR of mRNA isolated from axons and cell bodies of sympathetic neurons cultured in compartmentalised chambers. The absence of cell body material in axonal samples was assessed using primers amplifying *Histone H4* transcripts. Noncontiguous lanes from the same experiment and gels are shown side by side, as indicated by lines in the figure.

Fig. S6

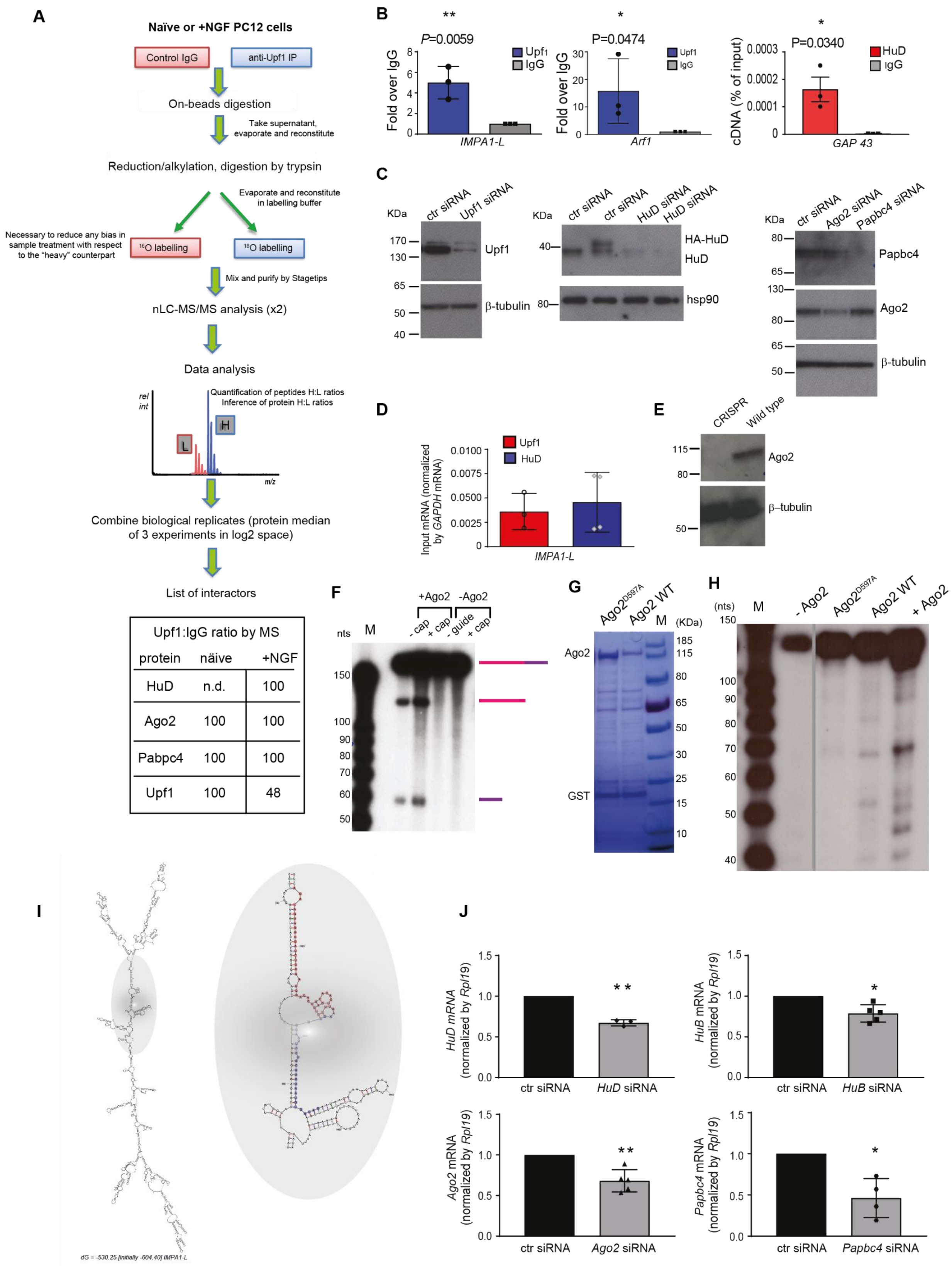

Figure S6. Remodeling of IMPA1 3'UTR by a complex that includes Ago2, HuD and Upf1. Related to Figure 6.

(**A**) Workflow employed for the discovery of Upf1 interactors in naïve and NGF-stimulated cells. The table shows the enrichment of the indicated interactors in the Upf1 immuno-precipitates of naïve or differentiated (+NGF) PC12 cells as measured by mass-spectrometry.

(**B**) RIP of *IMPA1-L* mRNA (*Left*), *Arf1* (as positive control for Upf1 antibody, *Middle*) or *GAP43* (as positive control for HuD antibody, *Right*) in sympathetic neurons. mRNAs were immunoprecipitated either with normal IgG, anti-Upf1 or anti-HuD antibodies, and subject to RT-qPCR. $\Delta\Delta$Ct values between antibody and IgG samples expressed as fold of IgG are shown. (*Left* and *Middle)* Unpaired one-tail t-test ** t=4.385, df=4, *t-2.18, df=4. (*Right)* Paired one-tail t-test, t=3.636, df=2.

(**C**) Western blotting of PC12 cells transfected with the indicated siRNAs and tested for Upf1 (*Left*), HuD (*Middle*) or Ago2 and Papbc4 (*Right*) expression. α tubulin and hsp90 were used as loading controls.

(**D**) Normalized expression levels of *IMPA1-L* mRNA in HuD or Upf1 in inputs of RIP samples shown in Fig. 4D and quantified by RT-qPCR. Unpaired two-tailed t test, no statistically significant (*n*=4).

(**E**) Western blot analysis of Ago2 in a PC12 cell clone in which Ago2 was deleted by CRISPR (*n*=3).

(**F**) Biological activity of recombinant human Ago2 tested by *in vitro* cleavage assay on *luciferase* RNA using a *luciferase* guide siRNA. Two fragments of the expected sizes (125 and 57 nts) are detected only in the samples containing Ago2 and guide siRNA. The lack of other fragments demonstrates that the preparation of recombinant Ago2 is devoid of contaminant RNAses. Irrelevant lanes have been removed.

(**G**) Coomassie staining of polyacrylamide gels to separate recombinant protein preparations of mouse wildtype (WT) and catalytic mutant (D597A) Ago2 (*n*=2).

(**H**) Enzymatic activity of the recombinant protein preparations was assessed in reactions containing RNA oligo, *IMPA1* guide siRNA and no cytoplasmic lysate (*n*=2). Noncontiguous lanes from the same experiment and auto radiographies are shown side by side while irrelevant lanes have been removed, as indicated by lines in the figure.

(**I**) Folding of the *IMPA1-L* transcript is modelled using RNAfold server. The enlarged area shows the position of the cleavage site in the middle of a loop structure.

(**J**) RT-qPCR of PC12 cells transfected with either *HuB*, *HuD*, *Ago2* or *Pabpc4* siRNA. Unpaired two-tail t-test, t=14.94 df=4, t=5.763 df=8, t=65.216, df=8, t=4.544 df=6, for HuD, HuB, Ago2 and Pabpc4 siRNA, respectively (*n*= 3 to 5 as indicated).

All data in this figure are presented as mean ± s.e.m. Tests are indicated in the legend and P values in figure.

Fig. S7

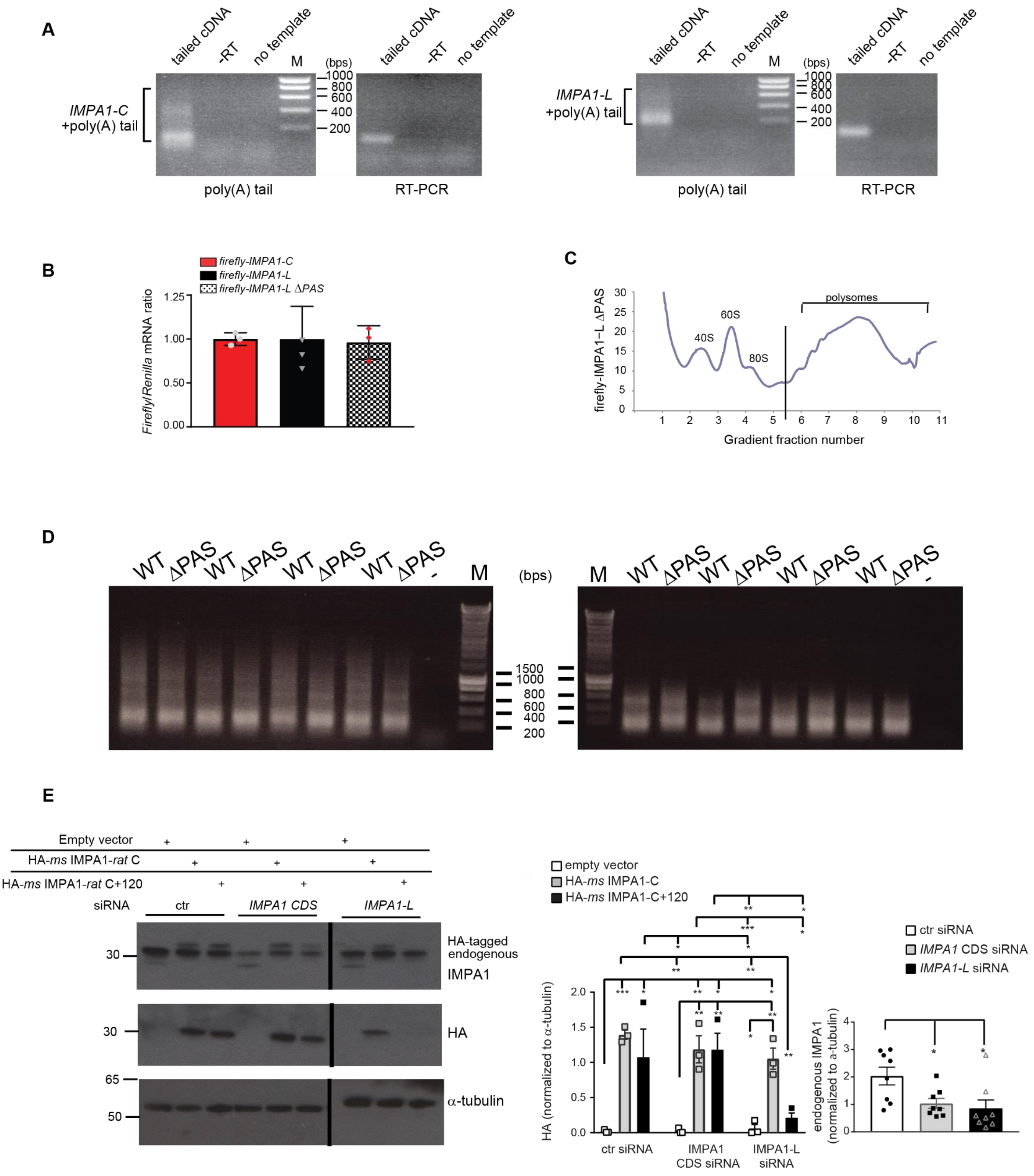

F

Construct	Transported to axons	Targeted by control siRNA	Targeted by IMPA1 total siRNA	Targeted by IMPA1L siRNA
Empty vector - EV	n.a.	×	×	×
HA-*ms* IMPA1-*rat* L	X	×	×	X
HA- *ms* IMPA1-*rat* C	×	×	×	×
HA-*ms* IMPA1-*rat* C + 120	X	×	×	X

n.a.= not applicable ×=no X=yes

Figure S7. *IMPA1-C* is polyadenylated and translated efficiently. Related to Figure 7.

(**A**) 3'end G/I tailing of mRNA isolated from PC12 cells and subjected to RT-PCR to assess poly(A) tail length of *IMPA1-C* (*Left*) or *IMPA1-L* (*Right*) 3'UTR. Noncontiguous lanes from

the same experiment and gels are shown side by side while irrelevant lanes have been removed, as indicated by the spacing in the figure (*n*=3).

(**B**) RT-qPCR analysis of *Firefly* and *Renilla luciferase* mRNAs isolated from PC12 cells transfected with the indicated vectors. One way Anova, non-statistically significative, df= 7 (*n*=3-4).

(**C**) Representative absorbance profile (A254nm) of polysomal fractions isolated from PC12 cells transfected with Firefly-IMPA1L-ΔPAS. Peaks representing the 40S, 60S and 80S ribosomal subunits, polysomal fractions and RNA granules are indicated. Line shows separation between free-monosomal and polysomal fractions.

(**D**) 3'end G/I tailing of mRNA isolated from PC12 cells transfected with firefly-IMPA1-L wild type or ΔPAS. Amplification was performed in nested PCR format to assess poly(A) tail length of the transcripts generated from the transfected expression vectors. Three independent samples are shown.

(**E**) (*Left*) IMPA1, hemagglutinin (HA) and α tubulin western blotting of PC12 cells transfected with the indicated siRNAs and vectors. In the IMPA1 blots, the higher band is HA-tagged mouse IMPA1 and the band under is endogenous rat IMPA1. Noncontiguous lanes from two blots from the same experiment are shown side by side, while irrelevant lanes have been removed, as indicated by the grey line in the figure (*n*=3). (*Right*) Densitometry analysis of the expression of HA-tagged IMPA1 or endogenous IMPA1 protein in PC12 cells transfected with the indicated siRNAs and vectors. HA and IMPA1 levels are normalized to α tubulin. One way Anova Tukey's post hoc test, *P<0.05, **P≤0.005, ***P≤0.00, df=18 (*n*=3-8).

(**F**) Table summarizing the subcellular localization and silencing of the vectors used in Figure 7A and B.

All data in this figure are presented as mean ± s.e.m. Tests are indicated in the legend and significant P values in figure.

Table S1. Related to Figure 4. List of proteins identified by mass spectrometry in immunoprecipitation experiments.

Accession	Description	Seq. Coverage	Σ# Proteins	Σ# Unique Peptides	Σ# Peptides	Σ# PSMs	Naive: CONT 1	Naive: CONT 2	Naive: CONT 3	NGF: CONT 1	NGF: CONT 2	NGF: CONT 3	Median Naive:CONT	Median NGF:CONT	Molecular Function	Cellular Component	Biological Process
A0A0A0MXX0	CD2-associated protein (Fragment) OS=Rattus norvegicus GN=Cd2ap PE=4 SV=1 - [A0A0A0MXX0_RAT]	35.32	2	18	18	126	100.0	100.0	100.0	100.0	100.0	100.0	100.0	100.0			
D4AB03	Protein Fam120a OS=Rattus norvegicus GN=Fam120a PE=4 SV=2 - [D4AB03_RAT]	16.14	1	13	13	41	93.6	100.0	100.0	100.0	100.0	100.0	100.0	100.0		cytoplasm	
Q6MG49	Large proline-rich protein BAG6 OS=Rattus norvegicus GN=Bag6 PE=2 SV=2 - [BAG6_RAT]	14.40	1	13	13	39	100.0	100.0	100.0	100.0	100.0	100.0	100.0	100.0	protein binding	nucleus; cytoplasm; cytosol	metabolic process; transport; cell death; cell organization and biogenesis; cell differentiation; regulation of biological process; response to stimulus
P62961	Nuclease-sensitive element-binding protein 1 OS=Rattus norvegicus GN=Ybx1 PE=2 SV=3 - [YBOX1_RAT]	21.12	10	3	4	21		68.0	100.0	100.0	100.0	100.0	82.5	100.0	DNA binding	nucleus; spliceosomal complex; cytoplasm	regulation of biological process
Q68A21	Transcriptional activator protein Pur-beta OS=Rattus norvegicus GN=Purb PE=1 SV=3 - [PURB_RAT]	16.19	3	3	5	19	9.7	100.0	74.6	100.0	29.7	100.0	74.6	100.0	translation regulator activity; DNA binding; RNA binding; protein binding	nucleus	metabolic process; regulation of biological process
G3V9N0	Polyadenylate-binding protein OS=Rattus norvegicus GN=Pabpc4 PE=2 SV=2 - [G3V9N0_RAT]	6.39	2	2	5	18	100.0	100.0	100.0	100.0	100.0		100.0	100.0	nucleotide binding; RNA binding		
D3ZB30	Polypyrimidine tract binding protein 1, isoform CRA_c OS=Rattus norvegicus GN=Ptbp1 PE=4 SV=1 - [D3ZB30_RAT]	16.98	4	7	7	18	100.0	100.0	61.5	100.0	100.0		100.0	100.0	nucleotide binding; DNA binding; RNA binding; catalytic activity	nucleus; membrane	regulation of biological process; metabolic process
Q3T1K0	Apolipoprotein B mRNA editing enzyme, catalytic polypeptide-like 3F OS=Rattus norvegicus GN=Apobec3b PE=2 SV=1 - [Q3T1K0_RAT]	9.87	1	3	3	13	100.0	100.0	100.0	100.0	100.0		100.0	100.0	catalytic activity; metal ion binding; RNA binding	nucleus; cytoplasm	cell differentiation; metabolic process; response to stimulus; regulation of biological process; defense response
D4A6A2	Heterogeneous nuclear ribonucleoprotein A3 OS=Rattus norvegicus GN=Hnrnpa3 PE=1 SV=2 - [D4A6A2_RAT]	11.60	7	3	3	12	0.1	100.0	100.0	100.0	100.0		100.0	100.0	nucleotide binding		
O09032	ELAV-like protein 4 OS=Rattus norvegicus GN=Elavl4 PE=1 SV=1 - [ELAV4_RAT]	18.23	1	2	6	11				100.0	100.0			100.0	nucleotide binding; RNA binding		
P62755	40S ribosomal protein S6 OS=Rattus norvegicus GN=Rps6 PE=1 SV=1 - [RS6_RAT]	19.84	2	4	5	9		100.0			100.0		100.0	100.0	structural molecule activity; protein binding	nucleus; cytoplasm; ribosome	metabolic process; regulation of biological process; response to stimulus
F1M5X1	Protein Rrbp1 OS=Rattus norvegicus GN=Rrbp1 PE=4 SV=2 - [F1M5X1_RAT]	3.88	5	4	4	9	100.0	100.0		100.0	100.0		100.0	100.0			transport
D3ZZ10	Protein Zcchc3 OS=Rattus norvegicus GN=Zcchc3 PE=4 SV=1 - [D3ZZ10_RAT]	15.75	1	5	5	9		82.2	100.0	100.0	100.0		90.7	100.0	metal ion binding; RNA binding		
B1H2A6	Fxr2 protein OS=Rattus norvegicus GN=Fxr2 PE=2 SV=1 - [B1H2A6_RAT]	14.07	1	4	6	9		100.0	100.0		100.0		100.0	100.0	RNA binding; protein binding	cytoplasm; membrane	
Q7TP98	Interleukin enhancer-binding factor 2 OS=Rattus norvegicus GN=Ilf2 PE=2 SV=1 - [ILF2_RAT]	5.83	1	2	2	8		83.6	100.0		100.0		91.4	100.0	DNA binding; RNA binding; nucleotide binding; catalytic activity	nucleus; cytoplasm; membrane	metabolic process; regulation of biological process; response to stimulus
F1LRP7	Protein argonaute-2 (Fragment) OS=Rattus norvegicus GN=Ago2 PE=3 SV=1 - [F1LRP7_RAT]	1.99	3	2	2	8	100.0	100.0	100.0		100.0		100.0	100.0	RNA binding; catalytic activity; protein binding	cytoplasm	regulation of biological process; metabolic process
F1LWX1	Protein LOC100910714 (Fragment) OS=Rattus norvegicus GN=LOC100910714 PE=3 SV=1 - [F1LWX1_RAT]	8.49	3	1	1	7	59.8	100.0	100.0		100.0		100.0	100.0	structural molecule activity	ribosome	metabolic process
P62268	40S ribosomal protein S23 OS=Rattus norvegicus GN=Rps23 PE=1 SV=3 - [RS23_RAT]	7.69	3	1	1	7		100.0			100.0		100.0	100.0	structural molecule activity	ribosome	metabolic process
Q6AY21	GTPase activating protein (SH3 domain) binding protein 2 OS=Rattus norvegicus GN=G3bp2 PE=2 SV=1 - [Q6AY21_RAT]	5.57	1	2	2	6		75.9	100.0		100.0		87.1	100.0	nucleotide binding; RNA binding	cytoplasm	transport
B3DMA1	Atxn2l protein OS=Rattus norvegicus GN=Atxn2l PE=2 SV=1 - [B3DMA1_RAT]	2.15	1	2	2	6		100.0	80.1		100.0		89.5	100.0	RNA binding	cytoplasm; membrane	metabolic process; regulation of biological process; cell organization and biogenesis
D4A321	DEAH (Asp-Glu-Ala-His) box polypeptide 38 (Predicted), isoform CRA_a OS=Rattus norvegicus GN=Dhx38 PE=4 SV=2 - [D4A321_RAT]	5.04	1	6	6	11		100.0	100.0		100.0		100.0	100.0	nucleotide binding; catalytic activity; RNA binding	membrane; spliceosomal complex	metabolic process
D4A9L2	Protein Srsf1 OS=Rattus norvegicus GN=Srsf1 PE=4 SV=1 - [D4A9L2_RAT]	8.06	1	2	2	7		100.0			100.0		100.0	100.0	nucleotide binding; RNA binding; protein binding	nucleus; spliceosomal complex	cell organization and biogenesis; regulation of biological process; metabolic process
Q794E4	Heterogeneous nuclear ribonucleoprotein F OS=Rattus norvegicus GN=Hnrnpf PE=1 SV=3 - [HNRPF_RAT]	6.27	1	1	2	6		75.8				100.0	75.8	100.0	nucleotide binding; RNA binding; protein binding	nucleus; spliceosomal complex; cytoplasm; membrane	metabolic process; regulation of biological process
G3V9N1	RCG21137 OS=Rattus norvegicus GN=Pgam5 PE=4 SV=1 - [G3V9N1_RAT]	9.38	3	3	3	5		100.0			100.0		100.0	100.0	catalytic activity; enzyme regulator activity; protein binding	mitochondrion; membrane	metabolic process; regulation of biological process; cell death
F1M013	Protein LOC100910109 (Fragment) OS=Rattus norvegicus GN=LOC100910109 PE=4 SV=2 - [F1M013_RAT]	13.58	18	3	3	5		6.7			100.0		6.7	100.0			
P62909	40S ribosomal protein S3 OS=Rattus norvegicus GN=Rps3 PE=1 SV=1 - [RS3_RAT]	8.64	1	2	2	4		100.0			100.0		100.0	100.0	DNA binding; RNA binding; structural molecule activity; catalytic activity; protein binding	nucleus; cytoplasm; cytosol; ribosome; membrane	metabolic process; response to stimulus; regulation of biological process
F1LXF5	Protein Get4 OS=Rattus norvegicus GN=Get4 PE=4 SV=2 - [F1LXF5_RAT]	11.52	1	3	3	3		64.1		100.0			64.1	100.0		cytosol	
P62850	40S ribosomal protein S24 OS=Rattus norvegicus GN=Rps24 PE=2 SV=1 - [RS24_RAT]	4.55	4	1	1	3		100.0			100.0		100.0	100.0	nucleotide binding; structural molecule activity	nucleus; cytoplasm; ribosome	metabolic process
H7C5Y5	60S ribosomal protein L6 OS=Rattus norvegicus GN=Rpl6 PE=3 SV=1 - [H7C5Y5_RAT]	2.69	3	1	1	2		9.9			100.0		9.9	100.0	structural molecule activity	ribosome	metabolic process
D4ADH2	Protein Pcbp1 (Fragment) OS=Rattus norvegicus GN=Pcbp1 PE=4 SV=2 - [D4ADH2_RAT]	4.12	3	1	1	2		100.0		100.0			100.0	100.0	RNA binding		
P62912	60S ribosomal protein L32 OS=Rattus norvegicus GN=Rpl32 PE=1 SV=2 - [RL32_RAT]	9.70	3	1	1	2		100.0			100.0		100.0	100.0	structural molecule activity	ribosome	metabolic process
D4A0L4	Y-box-binding protein 3 OS=Rattus norvegicus GN=Ybx3 PE=4 SV=2 - [D4A0L4_RAT]	10.27	8	1	2	2	81.5					100.0	81.5	100.0	DNA binding		regulation of biological process

Q9EPH8	Polyadenylate-binding protein 1 OS=Rattus norvegicus GN=Pabpc1 PE=1 SV=1 - [PABP1_RAT]	33.65	1	17	20	131	84.2	87.2	63.5	100.0	94.1	92.4	84.2	94.1	nucleotide binding; RNA binding; protein binding	nucleus; spliceosomal complex; cytoplasm; membrane	metabolic process; regulation of biological process
F2Z3R2	Protein Fblim1 OS=Rattus norvegicus GN=Hnrnpl PE=4 SV=1 - [F2Z3R2_RAT]	3.58	4	2	2	3	100.0				90.9		100.0	90.9	nucleotide binding; RNA binding; DNA binding	nucleus; membrane	metabolic process
D3ZR64	Protein Zfp598 OS=Rattus norvegicus GN=Zfp598 PE=4 SV=2 - [D3ZR64_RAT]	3.66	1	2	2	7		52.3	100.0		100.0	72.4	72.3	85.1	protein binding; metal ion binding; RNA binding		
P61980	Heterogeneous nuclear ribonucleoprotein K OS=Rattus norvegicus GN=Hnrnpk PE=1 SV=1 - [HNRPK_RAT]	17.71	3	9	9	21	83.8	64.2	100.0		62.5	100.0	83.8	79.1	DNA binding; RNA binding	nucleus; spliceosomal complex; cytoplasm; membrane	metabolic process; regulation of biological process
D3ZD73	Protein Ddx6 OS=Rattus norvegicus GN=Ddx6 PE=3 SV=1 - [D3ZD73_RAT]	2.90	1	1	1	4		100.0		53.3	85.5		100.0	67.5	nucleotide binding; catalytic activity; RNA binding	cytoplasm; membrane	metabolic process; cell organization and biogenesis
D3ZYW2	Heterogeneous nuclear ribonucleoprotein H OS=Rattus norvegicus GN=Hnrnph1 PE=4 SV=1 - [D3ZYW2_RAT]	16.32	4	5	6	24	100.0	100.0		0.3	63.5	100.0	100.0	63.5	nucleotide binding		
G3V6U4	ELAV-like protein OS=Rattus norvegicus GN=Elavl2 PE=3 SV=2 - [G3V6U4_RAT]	19.03	2	1	5	10	100.0			100.0	40.2		100.0	63.4	nucleotide binding; RNA binding		
P15865	Histone H1.4 OS=Rattus norvegicus GN=Hist1h1e PE=1 SV=3 - [H14_RAT]	19.20	5	6	6	55	73.8	52.6	13.7	35.4	60.8	100.0	52.6	60.8	DNA binding	chromosome; nucleus	cell organization and biogenesis
P60868	40S ribosomal protein S20 OS=Rattus norvegicus GN=Rps20 PE=3 SV=1 - [RS20_RAT]	19.33	3	2	2	7	100.0	58.9			51.3		76.7	51.3	RNA binding; structural molecule activity	cytoplasm; ribosome; membrane	metabolic process
P04177	Tyrosine 3-monooxygenase OS=Rattus norvegicus GN=Th PE=1 SV=3 - [TY3H_RAT]	22.29	1	9	9	53	0.4	86.3	19.0	51.0	8.0	100.0	19.0	51.0	catalytic activity; metal ion binding; protein binding	nucleus; cytoplasm; mitochondrion; endoplasmic reticulum; cytosol; membrane	response to stimulus; cell communication; metabolic process; regulation of biological process; transport; cell organization and biogenesis; cellular homeostasis
F1LY19	Protein Upf1 OS=Rattus norvegicus GN=Upf1 PE=4 SV=2 - [F1LY19_RAT] **BAIT**	47.69	2	48	48	292	23.5	100.0	100.0	48.3	53.7	33.8	100.0	48.3	DNA binding; catalytic activity; nucleotide binding; metal ion binding; RNA binding	chromosome; nucleus; cytoplasm; spliceosomal complex	metabolic process; response to stimulus; regulation of biological process
F1LPS8	Transcriptional activator protein Pur-alpha OS=Rattus norvegicus GN=Pura PE=4 SV=2 - [F1LPS8_RAT]	35.97	3	5	7	32	1.0	69.2	89.0	65.8	48.3	16.2	69.2	48.3	translation regulator activity; DNA binding; protein binding	nucleus; cytoplasm	metabolic process; regulation of biological process
B2GV38	Ubiquitin-like protein 4A OS=Rattus norvegicus GN=Ubl4a PE=2 SV=1 - [UBL4A_RAT]	25.48	1	4	4	22	100.0	100.0	100.0	48.1	100.0	8.0	100.0	48.1	protein binding	cytoplasm; cytosol	transport
P62856	40S ribosomal protein S26 OS=Rattus norvegicus GN=Rps26 PE=3 SV=3 - [RS26_RAT]	7.83	2	1	1	4		100.0			27.3		100.0	27.3	structural molecule activity	ribosome	metabolic process
Q80WE1	Fragile X mental retardation protein 1 homolog OS=Rattus norvegicus GN=Fmr1 PE=1 SV=2 - [FMR1_RAT]	16.36	1	5	7	19	100.0	100.0	100.0	3.4	100.0		100.0	18.5	RNA binding; protein binding	nucleus; cytoplasm; membrane	regulation of biological process; transport
Q5XI81	Fragile X mental retardation syndrome-related protein 1 OS=Rattus norvegicus GN=Fxr1 PE=2 SV=1 - [FXR1_RAT]	10.92	2	3	4	9		100.0			17.1		100.0	17.1	RNA binding	nucleus; cytoplasm; membrane	development; regulation of biological process; cell differentiation
P62243	40S ribosomal protein S8 OS=Rattus norvegicus GN=Rps8 PE=1 SV=2 - [RS8_RAT]	34.62	4	7	7	19	6.9	37.5	27.3	0.0	15.7	100.0	27.3	15.7	structural molecule activity; RNA binding	nucleus; cytoplasm; ribosome; membrane	metabolic process
P62919	60S ribosomal protein L8 OS=Rattus norvegicus GN=Rpl8 PE=2 SV=2 - [RL8_RAT]	10.51	2	2	2	9	8.7	20.7		4.7	47.8		13.4	15.0	RNA binding; structural molecule activity	ribosome	metabolic process
D3ZBX4	Ribosomal protein OS=Rattus norvegicus GN=RGD1559639 PE=3 SV=1 - [D3ZBX4_RAT]	13.36	2	2	2	5		7.0			3.9		7.0	3.9	RNA binding; structural molecule activity	ribosome	metabolic process
D3ZF34	Uncharacterized protein (Fragment) OS=Rattus norvegicus PE=4 SV=2 - [D3ZF34_RAT]	7.18	3	1	3	5		29.1			3.4		29.1	3.4	nucleotide binding		
M0RAR9	Uncharacterized protein (Fragment) OS=Rattus norvegicus PE=4 SV=1 - [M0RAR9_RAT]	7.27	3	1	3	4		100.0			3.4		100.0	3.4	nucleotide binding		
P06761	78 kDa glucose-regulated protein OS=Rattus norvegicus GN=Hspa5 PE=1 SV=1 - [GRP78_RAT]	4.59	1	3	3	8		5.9	0.4		3.2		1.6	3.2	nucleotide binding; protein binding; catalytic activity; enzyme regulator activity	nucleus; cytoplasm; mitochondrion; endoplasmic reticulum; organelle lumen;	cell organization and biogenesis; regulation of biological process; response to stimulus; cell communication; metabolic process
M0R6I6	Ribosomal protein L15 OS=Rattus norvegicus PE=3 SV=1 - [M0R6I6_RAT]	5.45	3	1	1	5		18.0		0.1	100.0		18.0	3.1	structural molecule activity	ribosome	metabolic process
P0CG51	Polyubiquitin-B OS=Rattus norvegicus GN=Ubb PE=1 SV=1 - [UBB_RAT]	52.46	9	4	4	25	0.9	3.3	1.1	5.1	2.4	1.6	1.1	2.4	protein binding	nucleus; cytoplasm	cell organization and biogenesis; cellular homeostasis
M0R757	Elongation factor 1-alpha OS=Rattus norvegicus GN=LOC100360413 PE=3 SV=1 - [M0R757_RAT]	11.47	4	4	4	19		2.5	0.6		1.5	1.5	1.2	1.5	nucleotide binding; RNA binding; catalytic activity; protein binding	nucleus; cytoplasm; membrane	metabolic process; response to stimulus
P21531	60S ribosomal protein L3 OS=Rattus norvegicus GN=Rpl3 PE=1 SV=3 - [RL3_RAT]	11.17	3	5	5	8	0.1	27.8		0.1	16.1		1.3	1.2	structural molecule activity; RNA binding	nucleus; cytoplasm; ribosome	metabolic process; response to stimulus
P63018	Heat shock cognate 71 kDa protein OS=Rattus norvegicus GN=Hspa8 PE=1 SV=1 - [HSP7C_RAT]	17.47	7	10	10	38	1.0	0.4	0.8	0.2	1.2	1.6	0.8	1.2	nucleotide binding		cell organization and biogenesis
G3V8L3	Lamin A, isoform CRA_b OS=Rattus norvegicus GN=Lmna PE=3 SV=1 - [G3V8L3_RAT]	1.50	2	1	1	7		1.3	0.6		1.0		0.9	1.0	structural molecule activity	nucleus; membrane; cytoplasm	cell organization and biogenesis; regulation of biological process; transport; response to stimulus
P00763	Anionic trypsin-2 OS=Rattus norvegicus GN=Prss2 PE=1 SV=2 - [TRY2_RAT]	4.12	5	1	1	14		0.8	1.0		0.7	1.1	0.9	0.9	catalytic activity		metabolic process
D3ZUC2	Protein Mov10 OS=Rattus norvegicus GN=Mov10 PE=4 SV=1 - [D3ZUC2_RAT]	3.98	1	3	3	3		98.4		0.7			98.4	0.7	nucleotide binding; catalytic activity; RNA binding		metabolic process
V9GZ85	Actin, cytoplasmic 2 (Fragment) OS=Rattus norvegicus GN=LOC100361457 PE=3 SV=1 - [V9GZ85_RAT]	12.03	3	2	4	19	0.6	4.8	0.6	0.4	0.9		0.6	0.6			
Q5BJT0	Arginine and glutamate-rich protein 1 OS=Rattus norvegicus GN=Arglu1 PE=2 SV=1 - [ARGL1_RAT]	13.65	1	5	5	15	0.0	0.8	0.8		0.5	0.7	0.8	0.6		nucleus; mitochondrion	cell communication; regulation of biological process; response to stimulus
Q4KLM7	Protein Specc1 OS=Rattus norvegicus GN=Specc1 PE=2 SV=1 - [Q4KLM7_RAT]	0.90	3	1	1	23	0.0	1.2	0.6			0.6	0.6	0.6	nucleotide binding		
P63269	Actin, gamma-enteric smooth muscle OS=Rattus norvegicus GN=Actg2 PE=2 SV=1 - [ACTH_RAT]	13.56	4	2	4	10	0.5	1.8	0.6	0.3	0.9		0.6	0.5	nucleotide binding	cytoplasm; cytoskeleton	
F1LMV6	Protein Dsp OS=Rattus norvegicus GN=Dsp PE=1 SV=1 - [F1LMV6_RAT]	5.07	1	16	16	32	0.0		0.0		0.5	0.3	0.0	0.4	catalytic activity; motor activity; protein binding; structural molecule activity; RNA binding	cytoskeleton; nucleus; cytoplasm; mitochondrion; membrane	cell organization and biogenesis; metabolic process; cell differentiation; response to stimulus; cell communication; regulation of biological process
D3ZFC3	Versican core protein (Fragment) OS=Rattus norvegicus GN=Vcan PE=4 SV=2 - [D3ZFC3_RAT]	1.18	3	1	1	6	0.3			0.4			0.1	0.4	metal ion binding; protein binding		
Q6P0K8	Junction plakoglobin OS=Rattus norvegicus GN=Jup PE=1 SV=1 - [PLAK_RAT]	11.41	1	7	7	20	0.1		0.0		0.1		0.1	0.1	structural molecule activity; protein binding	nucleus; cytoplasm; cytosol; cytoskeleton; membrane	regulation of biological process; cell organization and biogenesis; response to stimulus; cellular component movement; cell communication
P43244	Matrin-3 OS=Rattus norvegicus GN=Matr3 PE=1 SV=2 - [MATR3_RAT]	5.09	1	3	3	4	54.7	100.0	100.0				100.0		nucleotide binding; RNA binding; metal ion binding	nucleus; membrane	
P34058	Heat shock protein HSP 90-beta OS=Rattus norvegicus GN=Hsp90ab1 PE=1 SV=4 - [HS90B_RAT]	5.25	2	3	3	4	3.0	18.7	0.7				3.0		nucleotide binding; RNA binding; protein binding	cytoplasm; mitochondrion; cytosol; cell surface; membrane	metabolic process; response to stimulus; regulation of biological process; cell organization and biogenesis
D4A533	Protein Tapt1 (Fragment) OS=Rattus norvegicus GN=Tapt1 PE=4 SV=2 - [D4A533_RAT]	5.03	1	1	1	2	71.3	100.0					84.5				

Table S2. Related to Figure 4. Putative interactors of Upf1 were singled out according to the following criteria: (i) identified and quantified in at least two replicate experiments;

(ii) fold change Upf1 IP:IgG control > 2 in all available replicates.

Accession	Description	Seq. Coverage	Σ# Proteins	Σ# Unique Peptides	Σ# Peptides	Σ# PSMs	Naive: CONT 1	Naive: CONT 2	Naive: CONT 3	NGF: CONT 1	NGF: CONT 2	NGF: CONT 3	Median Naive:CONT	Median NGF:CONT	Molecular Function	Cellular Component	Biological Process
F1LY19	Protein Upf1 OS=Rattus norvegicus GN=Upf1 PE=4 SV=2 - [F1LY19_RAT] **BAIT**	47.69	2	48	48	292	23.5	100.0	100.0	48.3	53.7	33.8	100.0	48.3	DNA binding; catalytic activity; nucleotide binding; metal ion binding; RNA binding	chromosome; nucleus; cytoplasm; spliceosomal complex	metabolic process; response to stimulus; regulation of biological process
Q9EPH8	Polyadenylate-binding protein 1 OS=Rattus norvegicus GN=Pabpc1 PE=1 SV=1 - [PABP1_RAT]	33.65	1	17	20	131	84.2	87.2	63.5	100.0	94.1	92.4	84.2	94.1	nucleotide binding; RNA binding; protein binding	nucleus; spliceosomal complex; cytoplasm; membrane	metabolic process; regulation of biological process
A0A0A0MXX0	CD2-associated protein (Fragment) OS=Rattus norvegicus GN=Cd2ap PE=4 SV=1 - [A0A0A0MXX0_RAT]	35.32	2	18	18	126	100.0	100.0	100.0	100.0	100.0	100.0	100.0	100.0			
P15865	Histone H1.4 OS=Rattus norvegicus GN=Hist1h1e PE=1 SV=3 - [H14_RAT]	19.20	5	6	6	55	73.8	52.6	13.7	35.4	60.8	100.0	52.6	60.8	DNA binding	chromosome; nucleus	cell organization and biogenesis
D4AB03	Protein Fam120a OS=Rattus norvegicus GN=Fam120a PE=4 SV=2 - [D4AB03_RAT]	16.14	1	13	13	41	93.6	100.0	100.0	100.0	100.0	100.0	100.0	100.0		cytoplasm	
Q6MG49	Large proline-rich protein BAG6 OS=Rattus norvegicus GN=Bag6 PE=2 SV=2 - [BAG6_RAT]	14.40	1	13	13	39	100.0	100.0	100.0	100.0	100.0	100.0	100.0	100.0	protein binding	nucleus; cytoplasm; cytosol	metabolic process; transport; cell death; cell organization and biogenesis; cell differentiation; regulation of biological process; response to stimulus
B2GV38	Ubiquitin-like protein 4A OS=Rattus norvegicus GN=Ubl4a PE=2 SV=1 - [UBL4A_RAT]	25.48	1	4	4	22	100.0	100.0	100.0	48.1	100.0	8.0	100.0	48.1	protein binding	cytoplasm; cytosol	transport
P62961	Nuclease-sensitive element-binding protein 1 OS=Rattus norvegicus GN=Ybx1 PE=2 SV=3 - [YBOX1_RAT]	21.12	10	3	4	21		68.0	100.0	100.0	100.0	100.0	82.5	100.0	DNA binding	nucleus; spliceosomal complex; cytoplasm	regulation of biological process
P61980	Heterogeneous nuclear ribonucleoprotein K OS=Rattus norvegicus GN=Hnrnpk PE=1 SV=1 - [HNRPK_RAT]	17.71	3	9	9	21	83.8	64.2	100.0		62.5	100.0	83.8	79.1	DNA binding; RNA binding	nucleus; spliceosomal complex; cytoplasm; membrane	metabolic process; regulation of biological process
Q68A21	Transcriptional activator protein Pur-beta OS=Rattus norvegicus GN=Purb PE=1 SV=3 - [PURB_RAT]	16.19	3	3	5	19	9.7	100.0	74.6	100.0	29.7	100.0	74.6	100.0	translation regulator activity; DNA binding; RNA binding; protein binding	nucleus	metabolic process; regulation of biological process
Q80WE1	Fragile X mental retardation protein 1 homolog OS=Rattus norvegicus GN=Fmr1 PE=1 SV=2 - [FMR1_RAT]	16.36	1	5	7	19	100.0	100.0	100.0	3.4	100.0		100.0	18.5	RNA binding; protein binding	nucleus; cytoplasm; membrane	regulation of biological process; transport
G3V9N0	Polyadenylate-binding protein OS=Rattus norvegicus GN=Pabpc4 PE=2 SV=2 - [G3V9N0_RAT]	6.39	2	2	5	18	100.0	100.0	100.0	100.0	100.0		100.0	100.0	nucleotide binding; RNA binding		
D3ZB30	Polypyrimidine tract binding protein 1, isoform CRA_c OS=Rattus norvegicus GN=Ptbp1 PE=4 SV=1 - [D3ZB30_RAT]	16.98	4	7	7	18	100.0	100.0	61.5	100.0	100.0		100.0	100.0	nucleotide binding; DNA binding; RNA binding; catalytic activity	nucleus; membrane	regulation of biological process; metabolic process
Q3T1K0	Apolipoprotein B mRNA editing enzyme, catalytic polypeptide-like 3F OS=Rattus norvegicus GN=Apobec3b PE=2 SV=1 - [Q3T1K0_RAT]	9.87	1	3	3	13	100.0	100.0	100.0	100.0	100.0		100.0	100.0	catalytic activity; metal ion binding; RNA binding	nucleus; cytoplasm	cell differentiation; metabolic process; response to stimulus; regulation of biological process; defense response
O09032	ELAV-like protein 4 OS=Rattus norvegicus GN=Elavl4 PE=1 SV=1 - [ELAV4_RAT]	18.23	1	2	6	11				100.0	100.0			100.0	nucleotide binding; RNA binding		
D4A321	DEAH (Asp-Glu-Ala-His) box polypeptide 38 (Predicted), isoform CRA_a OS=Rattus norvegicus GN=Dhx38 PE=4 SV=2 - [D4A321_RAT]	5.04	1	6	6	11		100.0	100.0		100.0		100.0	100.0	nucleotide binding; catalytic activity; RNA binding	membrane; spliceosomal complex	metabolic process
G3V6U4	ELAV-like protein OS=Rattus norvegicus GN=Elavl2 PE=3 SV=2 - [G3V6U4_RAT]	19.03	2	1	5	10	100.0			100.0	40.2		100.0	63.4	nucleotide binding; RNA binding		
P62755	40S ribosomal protein S6 OS=Rattus norvegicus GN=Rps6 PE=1 SV=1 - [RS6_RAT]	19.84	2	4	5	9		100.0			100.0		100.0	100.0	structural molecule activity; protein binding	nucleus; cytoplasm; ribosome	metabolic process; regulation of biological process; response to stimulus
F1M5X1	Protein Rrbp1 OS=Rattus norvegicus GN=Rrbp1 PE=4 SV=2 - [F1M5X1_RAT]	3.88	5	4	4	9	100.0	100.0		100.0	100.0		100.0	100.0			transport
D3ZZ10	Protein Zcchc3 OS=Rattus norvegicus GN=Zcchc3 PE=4 SV=1 - [D3ZZ10_RAT]	15.75	1	5	5	9		82.2	100.0	100.0	100.0		90.7	100.0	metal ion binding; RNA binding		
B1H2A6	Fxr2 protein OS=Rattus norvegicus GN=Fxr2 PE=2 SV=1 - [B1H2A6_RAT]	14.07	1	4	6	9		100.0	100.0		100.0		100.0	100.0	RNA binding; protein binding	cytoplasm; membrane	
Q5XI81	Fragile X mental retardation syndrome-related protein 1 OS=Rattus norvegicus GN=Fxr1 PE=2 SV=1 - [FXR1_RAT]	10.92	2	3	4	9		100.0			17.1		100.0	17.1	RNA binding	nucleus; cytoplasm; membrane	development; regulation of biological process; cell differentiation
P62919	60S ribosomal protein L8 OS=Rattus norvegicus GN=Rpl8 PE=2 SV=2 - [RL8_RAT]	10.51	2	2	2	9	8.7	20.7		4.7	47.8		13.4	15.0	RNA binding; structural molecule activity	ribosome	metabolic process
Q7TP98	Interleukin enhancer-binding factor 2 OS=Rattus norvegicus GN=Ilf2 PE=2 SV=1 - [ILF2_RAT]	5.83	1	2	2	8		83.6	100.0		100.0		91.4	100.0	DNA binding; RNA binding; nucleotide binding; catalytic activity	nucleus; cytoplasm; membrane	metabolic process; regulation of biological process; response to stimulus
F1LRP7	Protein argonaute-2 (Fragment) OS=Rattus norvegicus GN=Ago2 PE=3 SV=1 - [F1LRP7_RAT]	1.99	3	2	2	8	100.0	100.0	100.0		100.0		100.0	100.0	RNA binding; catalytic activity; protein binding	cytoplasm	regulation of biological process; metabolic process
F1LWX1	Protein LOC100910714 (Fragment) OS=Rattus norvegicus GN=LOC100910714 PE=3 SV=1 - [F1LWX1_RAT]	8.49	3	1	1	7	59.8	100.0	100.0		100.0		100.0	100.0	structural molecule activity	ribosome	metabolic process
P62268	40S ribosomal protein S23 OS=Rattus norvegicus GN=Rps23 PE=1 SV=3 - [RS23_RAT]	7.69	3	1	1	7		100.0			100.0		100.0	100.0	structural molecule activity	ribosome	metabolic process
D4A9L2	Protein Srsf1 OS=Rattus norvegicus GN=Srsf1 PE=4 SV=1 - [D4A9L2_RAT]	8.06	1	2	2	7		100.0			100.0		100.0	100.0	nucleotide binding; RNA binding; protein binding	nucleus; spliceosomal complex	cell organization and biogenesis; regulation of biological process; metabolic process
D3ZR64	Protein Zfp598 OS=Rattus norvegicus GN=Zfp598 PE=4 SV=2 - [D3ZR64_RAT]	3.66	1	2	2	7		52.3	100.0		100.0	72.4	72.3	85.1	protein binding; metal ion binding; RNA binding		
P60868	40S ribosomal protein S20 OS=Rattus norvegicus GN=Rps20 PE=3 SV=1 - [RS20_RAT]	19.33	3	2	2	7	100.0	58.9			51.3		76.7	51.3	RNA binding; structural molecule activity	cytoplasm; ribosome; membrane	metabolic process
Q6AY21	GTPase activating protein (SH3 domain) binding protein 2 OS=Rattus norvegicus GN=G3bp2 PE=2 SV=1 - [Q6AY21_RAT]	5.57	1	2	2	6		75.9	100.0		100.0		87.1	100.0	nucleotide binding; RNA binding	cytoplasm	transport

B3DMA1	Atxn2l protein OS=Rattus norvegicus GN=Atxn2l PE=2 SV=1 - [B3DMA1_RAT]	2.15	1	2	2	6		100.0	80.1		100.0		89.5	100.0	RNA binding	cytoplasm; membrane	metabolic process; regulation of biological process; cell organization and biogenesis
Q794E4	Heterogeneous nuclear ribonucleoprotein F OS=Rattus norvegicus GN=Hnrnpf PE=1 SV=3 - [HNRPF_RAT]	6.27	1	1	2	6		75.8				100.0	75.8	100.0	nucleotide binding; RNA binding; protein binding	nucleus; spliceosomal complex; cytoplasm; membrane	metabolic process; regulation of biological process
G3V9N1	RCG21137 OS=Rattus norvegicus GN=Pgam5 PE=4 SV=1 - [G3V9N1_RAT]	9.38	3	3	3	5		100.0			100.0		100.0	100.0	catalytic activity; enzyme regulator activity; protein binding	mitochondrion; membrane	metabolic process; regulation of biological process; cell death
F1M013	Protein LOC100910109 (Fragment) OS=Rattus norvegicus GN=LOC100910109 PE=4 SV=2 - [F1M013_RAT]	13.58	18	3	3	5		6.7			100.0		6.7	100.0			
D3ZBX4	Ribosomal protein OS=Rattus norvegicus GN=RGD1559639 PE=3 SV=1 - [D3ZBX4_RAT]	13.36	2	2	2	5		7.0			3.9		7.0	3.9	RNA binding; structural molecule activity	ribosome	metabolic process
D3ZF34	Uncharacterized protein (Fragment) OS=Rattus norvegicus PE=4 SV=2 - [D3ZF34_RAT]	7.18	3	1	3	5		29.1			3.4		29.1	3.4	nucleotide binding		
P62909	40S ribosomal protein S3 OS=Rattus norvegicus GN=Rps3 PE=1 SV=1 - [RS3_RAT]	8.64	1	2	2	4		100.0			100.0		100.0	100.0	DNA binding; RNA binding; structural molecule activity; catalytic activity; protein binding	nucleus; cytoplasm; cytosol; ribosome; membrane	metabolic process; response to stimulus; regulation of biological process
D3ZD73	Protein Ddx6 OS=Rattus norvegicus GN=Ddx6 PE=3 SV=1 - [D3ZD73_RAT]	2.90	1	1	1	4		100.0		53.3	85.5		100.0	67.5	nucleotide binding; catalytic activity; RNA binding	cytoplasm; membrane	metabolic process; cell organization and biogenesis
P62856	40S ribosomal protein S26 OS=Rattus norvegicus GN=Rps26 PE=3 SV=3 - [RS26_RAT]	7.83	2	1	1	4		100.0			27.3		100.0	27.3	structural molecule activity	ribosome	metabolic process
M0RAR9	Uncharacterized protein (Fragment) OS=Rattus norvegicus PE=4 SV=1 - [M0RAR9_RAT]	7.27	3	1	3	4		100.0			3.4		100.0	3.4	nucleotide binding		
F1LXF5	Protein Get4 OS=Rattus norvegicus GN=Get4 PE=4 SV=2 - [F1LXF5_RAT]	11.52	1	3	3	3		64.1		100.0			64.1	100.0		cytosol	
P62850	40S ribosomal protein S24 OS=Rattus norvegicus GN=Rps24 PE=2 SV=1 - [RS24_RAT]	4.55	4	1	1	3		100.0			100.0		100.0	100.0	nucleotide binding; structural molecule activity	nucleus; cytoplasm; ribosome	metabolic process
F2Z3R2	Protein Fblim1 OS=Rattus norvegicus GN=Hnrnpl PE=4 SV=1 - [F2Z3R2_RAT]	3.58	4	2	2	3	100.0				90.9		100.0	90.9	nucleotide binding; RNA binding; DNA binding	nucleus; membrane	metabolic process
H7C5Y5	60S ribosomal protein L6 OS=Rattus norvegicus GN=Rpl6 PE=3 SV=1 - [H7C5Y5_RAT]	2.69	3	1	1	2		9.9			100.0		9.9	100.0	structural molecule activity	ribosome	metabolic process
D4ADH2	Protein Pcbp1 (Fragment) OS=Rattus norvegicus GN=Pcbp1 PE=4 SV=2 - [D4ADH2_RAT]	4.12	3	1	1	2		100.0		100.0			100.0	100.0	RNA binding		
P62912	60S ribosomal protein L32 OS=Rattus norvegicus GN=Rpl32 PE=1 SV=2 - [RL32_RAT]	9.70	3	1	1	2		100.0			100.0		100.0	100.0	structural molecule activity	ribosome	metabolic process
D4A0L4	Y-box-binding protein 3 OS=Rattus norvegicus GN=Ybx3 PE=4 SV=2 - [D4A0L4_RAT]	10.27	8	1	2	2	81.5					100.0	81.5	100.0	DNA binding		regulation of biological process

Table S3. Related to Figure 4. MS/MS spectra of protein entries identified by a single peptide match

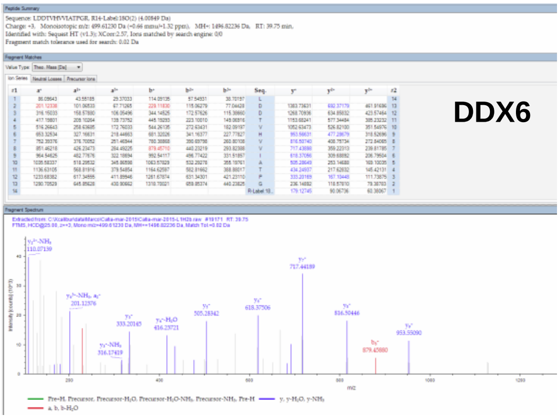

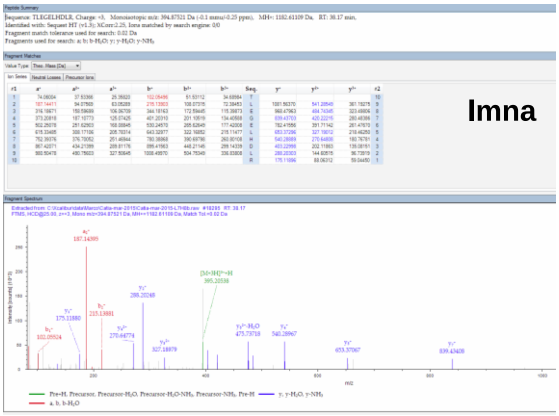

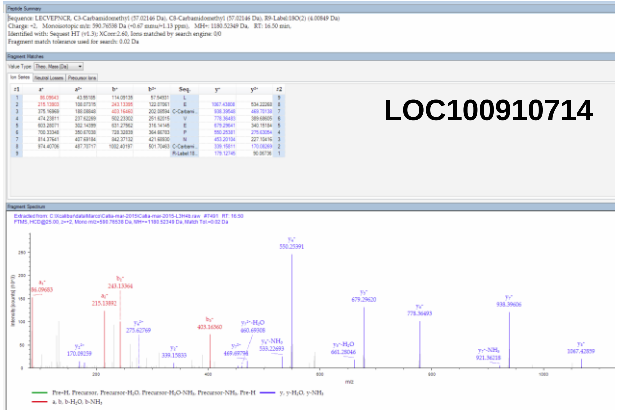

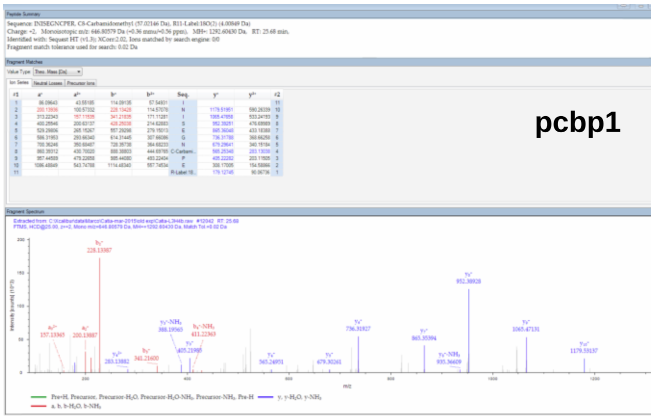

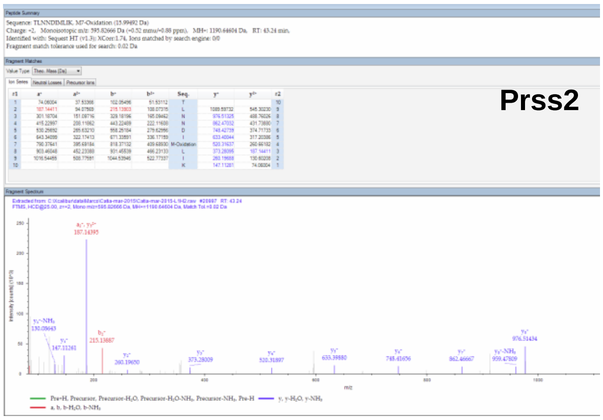

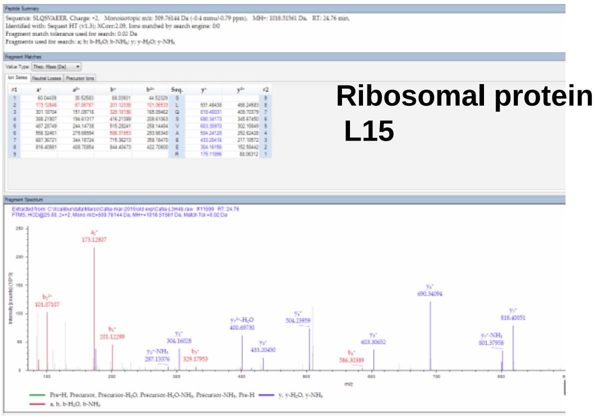

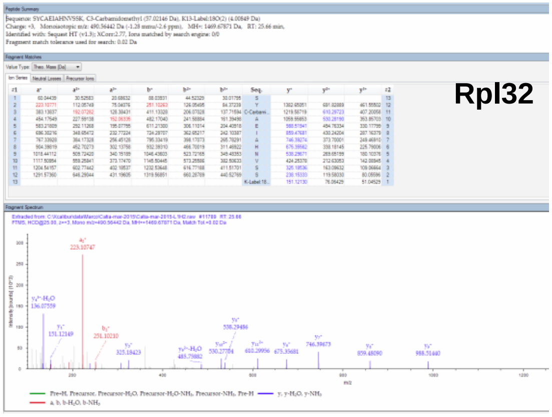

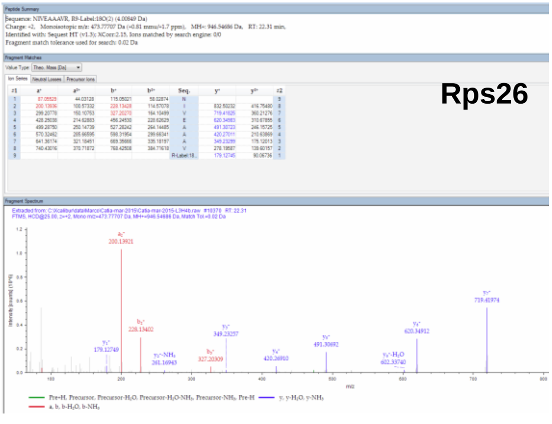

Rpl6

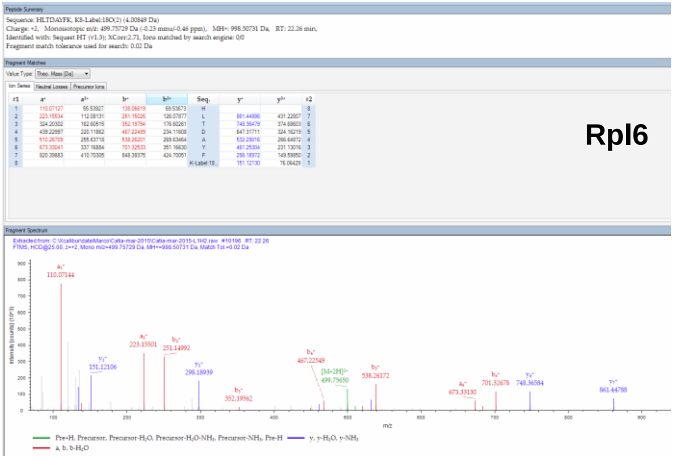

Rps23

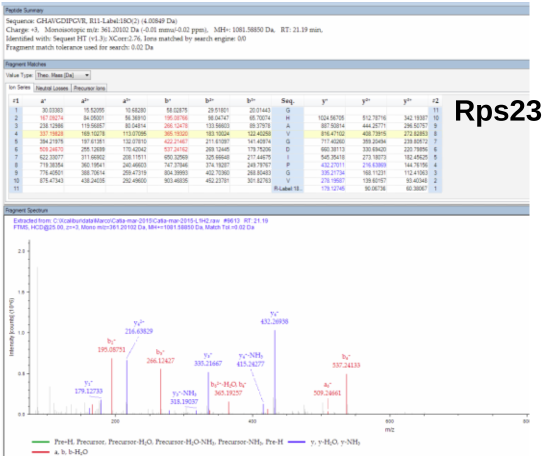

Rps24

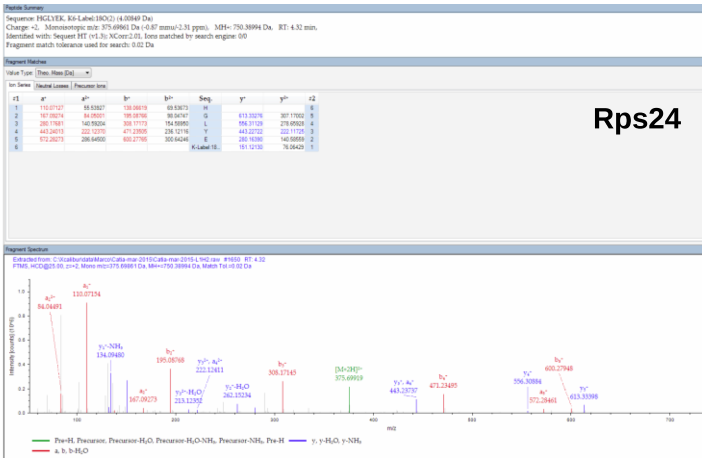

Specc1

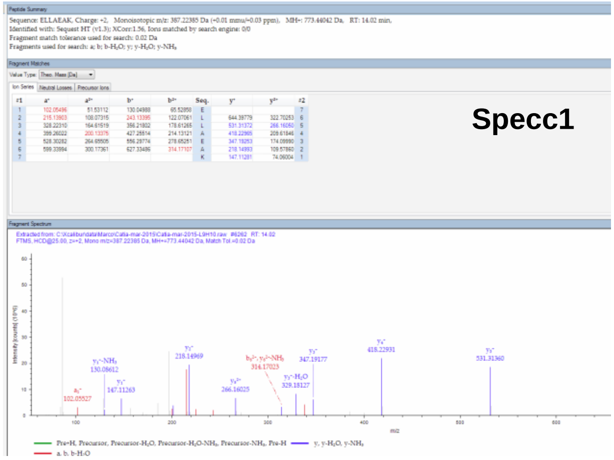

Tapt1

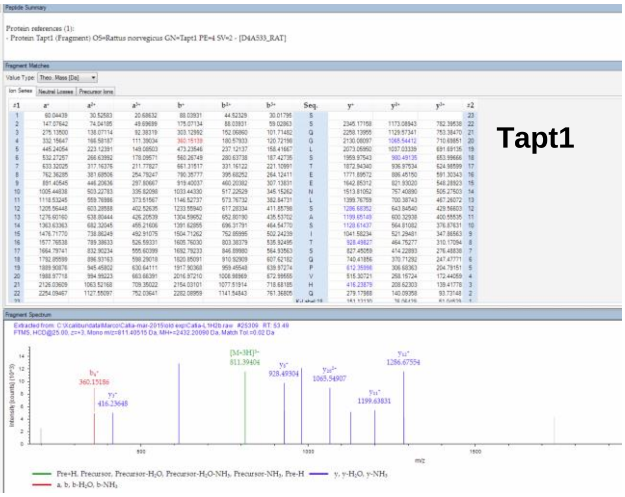

Vcan

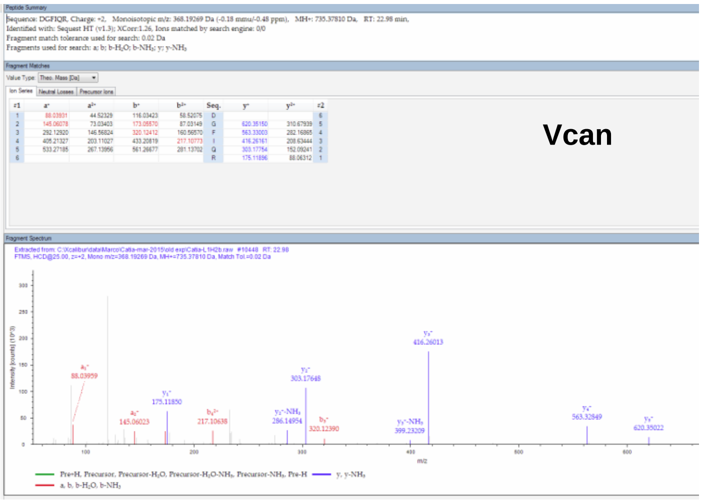

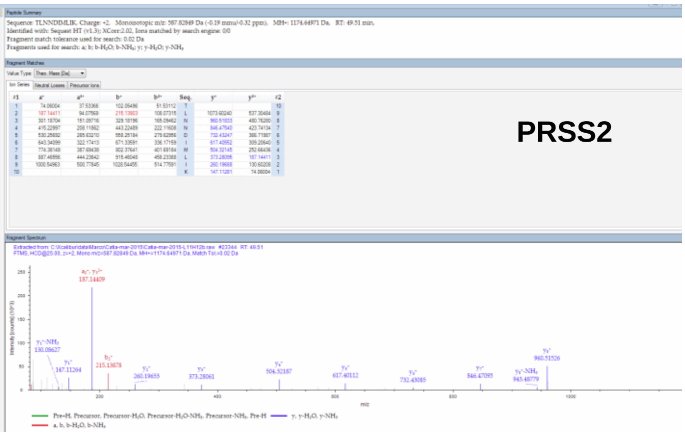

PRSS2

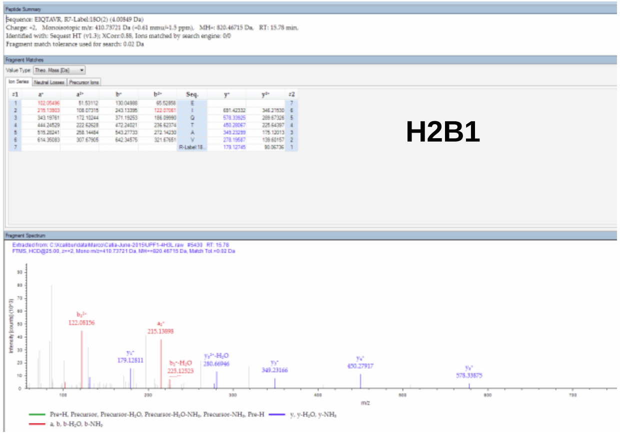

H2B1

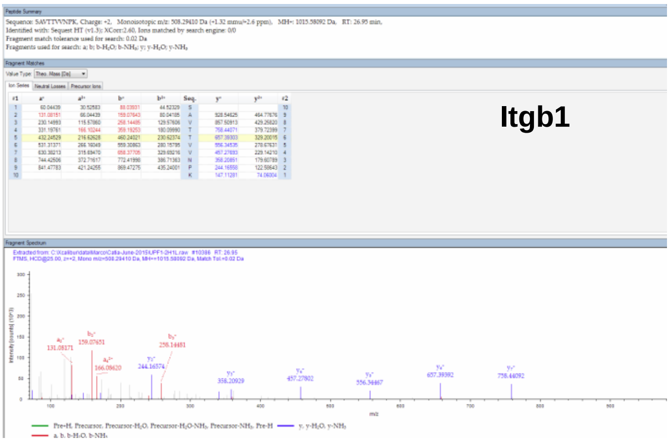

Itgb1

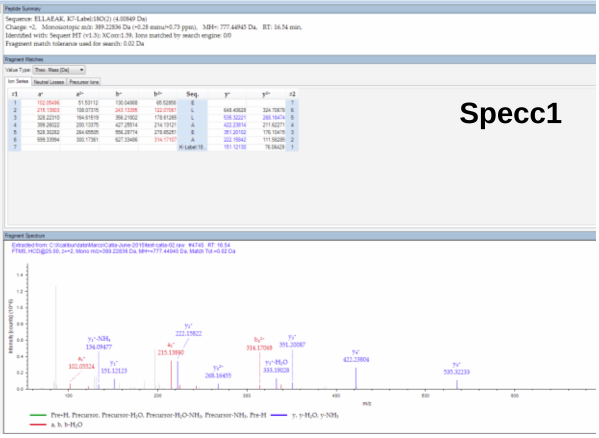

Specc1

Table S4: Related to STAR Methods. Primer and RNA oligos sequences, PCR conditions and antibodies.

Primer and RNA oligos names	Primer and RNA oligos sequences	NCBI Accession number	[Mg^{++}]	Tann.	Application
Rat Ago2 crRNA	CGUUACACGAUGCACUUUCGGUUUUAGAGCUAUG CU	NM_021597.1			CRISPR mutagenesis
Ago2 2358F<br>Ago2 2564R	CCAGTTCCAGCAGGTTCTTCA<br>GGTGGGTGATCTTTGTGTCGA	NM_021597.1	a	a	qPCR
Ago2 D597A Fwd<br>Ago2 D597A Rev	CTTCCTGGGAGCCGCCGTCACCCACCCACC<br>GGTGGGTGGGTGACGGCGGCTCCCAGGAAG	NM_021597.1	a	a	Mutagenesis PCR
rat Arf1-125 Fwd<br>rat Arf1-333 Rev	ATGCGCATTCTCATGGTGG<br>CTTGGGTGTTCTGGAAGTAGTGG	NM_022518.3	a	a	qPCR
R bact 82-F<br>R bact 292-R	ATG GAT GAC GAT ATC GCT GCG<br>GGT GAC AAT GCC GTG TTC AAT	NM_031144.3	2.5mM	56ºC	RT-PCR
b actin680 Fwd RACE	CAC CAC CAC AGC TGA GAG GGA AAT CGT GC	NM_031144	2.5mM	68°C b	RACE
Cofilin 86-Fwd RACE	CTCTGGTGTGGCTGTCTCTGATGG	NM_017147	2.5mM	68°C b	RACE
RAT CPSF3 1815F<br>RAT CPSF3 1956R	CATGTATGCAGACACAGTGACCACC<br>ATGTCCTGGAGCATGACCTCAAGC	NM_001030030.1	2.5mM	56°C	RT-PCR
BamHI Firefly_F<br>NotI Firefly_R	GCG TAA GGA TCC ATG GAA GAC GCC AAA AAC AT<br>GCC TGA AGC GGC CGC TTA CAC GGC GAT CTT TCC GC		1.5mM	65ºC	Cloning by PCR
Firefly-1385 F<br>Firefly-1560 R	F AAC ATC TTC GAC GCA GGT GTC G<br>CGT CCA CAA ACA CAA CTC CTC CG		a	58ºC	qPCR
Firefly-1601 F<br>UPM	CCGAAAGGTCTTACCGGAAAACTC<br>Proprietary (from the RACE kit)		a	58ºC	TA-PAT assay 1st amplification
GAPDH 898-F<br>GAPDH 1106-Rev	CACTGAGGACCAGGTTGTCTCC<br>GCCTCTCTCTTGCTCTCAGTATCC	NM_017008.4	a	58ºC	qPCR
GFP F2<br>GFP R2	GACGTAAACGGCCACAAGTT<br>AAGTCGTGCTGCTTCATGTG		a	60ºC	qPCR
HA 954F<br>ms IMPA1-1066R	CGCGAGATAGGATCCATGTACCCATACG<br>TCATCTCTCCAGCTTGCCTCG		a	60ºC	qPCR
HistoneH4 108–Fwd<br>HistoneH4 337–Rev	ACGCCTGTGGTCTTCAATCAGG<br>GCGGGTCTCCTCGTAGATGAG	M27433	2.5mM	56°C	RT-PCR
HuB 621F<br>HuB 765R T	AGCGGTTCGTGGGCAGCAGA<br>CCCGGAGTCAACTGGTGAGGAGC	NM_001302217.1	a	a	qPCR
HuD 304 F<br>HuD 464 R T	GGAGTCTCTTTGGGAGCATTGG<br>GGTTTTGGTCTGGAGCTGAGTCC	NM_001077651.2	a	a	qPCR
IMPA1 guide siRNA	[Phos]GAGGUGUCGAUUGGAGAUCGC				*In vitro* cleavage assay
IMPA 782F	TGCTGCTGGATGTGACAGGTGG	NM_032057.2	a	touchdown	3'RACE
IMPA1-1276F<br>UPM	CTGTAAGGCTGTAACCAGCAGGCTC<br>Proprietary (from the RACE kit)		a	58ºC	TA-PAT assay nested amplification
IMPA1 2027F<br>UPM	CTCTGATTTGGACCGTGCAATTAC<br>Proprietary (from the RACE kit)		a	58ºC	TA-PAT assay nested amplification
IMPA C2734t as<br>IMPA C2734t	CAA AGA GAG CAG TGG TGA AAT TCA GCA AGT TTT GAA AGA AAT CAA AA<br>TTT TGA TTT CTT TCA AAA CTT GCT GAA TTT CAC CAC TGC TCT CTT TG	NM_032057.2	a	55ºC	Mutagenesis PCR
impa-2045F<br>impa2165R	TGA TTT GGA CCG TGC AAT TAC<br>GGC TTG TAA GTA AAT AAA TTT AAT TGC	GU441530	a	58ºC	qPCR
IMPA L 1807 F<br>IMPA L 2039R	AAG GTC TTG GGC CTC TCA AA<br>TTG CAC GGT CCA AAT CAG AG	GU441530	a	a	qPCR
Luc guide siRNA	[Phos]UCGAAGUACUCAGCGUAAGUG				*In vitro* cleavage assay
LUC1 F<br>LUC1 R	TTG TGC CAG AGT CCT TCG AT<br>TAG GAT CTC TGG CAT GCG AG		a	60ºC	qPCR
mCherry 190F<br>mCherry 386R	GAG GGC ACC CAG ACC GCC AA<br>ACG CCG CCG TCC TCG AAG TT		a	60ºC	qPCR
myc 1612F<br>myc 1730R	CCAGGTCCTCAGACACCGAGG<br>CAACTCAGGGATCTGGTCGCG		a	60ºC	qPCR
Pabpc4 1927F<br>Pabpc4 2019R	GGCTCCATACAAGTATGCCTCCA<br>GACCCTGGACATGGACTGCA	NM_001100538.1	a	a	qPCR

pcDNA t356g<br>pcDNA t356g as	CAGCTGGGGCTCGAGGGGGTATCCC<br>GGGATACCCCCTCGAGCCCCAGCTG		a	55ºC	Mutagenesis PCR
Renilla-1072 Fwd<br>Renilla-1247 Rev	GAT GAT AAC TGG TCC GCA GTG G<br>GCG CTA CTG GCT CAA TAT GTG G		a	58ºC	qPCR
RML RNA oligo	CGACUGGAGCACGAGGACACUGACAUGGACUGAA GGAGUAGAAA				RNA ligation
RLM GR 5'<br>RLM IMPA 1743R	CGACTGGAGCACGAGGACACTGA<br>GGTCACACCAGCCAACACCACGGTATGG	NM_032057	a	72ºC	RLM RT-PCR
RLM GR 5'<br>RLM IMPA 1663R	CGACTGGAGCACGAGGACACTGA<br>ACC AGG TAC ATG GAA GAC GTC TGT GG	NM_032057	a	a	qPCR
RLM Sms 1984 R	CCTGAAGTCTAGTGTGCACTTGCACATCC	c	a	72ºC	RLM RT
RLM Maoa 2013R<br>RLM Nested Maoa 1968R	AGTGCCAAGGGTAGTGTGTATCACATGG<br>CCTCTGTACACCTTGGACGCTACAC	c	a	68°C	RLM RT<br>RLM RT-PCR
RLM Fdxr 1816R<br>Fdxr 1501F	GGTAGGGTCTGTCCGTACCTCCATCC<br>TGGTGGATCGAAGAGAGATGCTGCAGC	NM_024153.1	a	72ºC	RLM RT<br>RT-PCR
RLM Maf1 1267R<br>Maf1 1048F	TGGACATCCACAGGCTGAAACCAAAGG<br>GAGAGTGGAGGTGGAGGTGGAGG	NM_001014085.1	a	72ºC<br>68C	RLM RT<br>RT-PCR
RLM Cops3 1441R<br>Cops3 1125F	GCGTGGACCAGCATGGTACTTCC<br>CCAGAAGGATGGTATGGTCAGTTTCC	NM_001004200.1	a	72ºC<br>68ºC	RLM RT<br>RT-PCR
Rpl19 F<br>Rpl19 R	GGA TGC GAA GGA TGA GGA T<br>CCA TGA GAA TCC GCT TGT TT	NM_031103.1	a	a	qPCR
Rpl10a 361F<br>Rpl10a 558R	GACCTCCGAGTCTCTGATCAAGC<br>CGTCATCGGTCATCTTCACGTGG			60°C	qPCR
Stathmin 2 377 Fwd RACE	ACTTCAGCAAGATGGCGGAGGAGAA	NM_053440.2	2.5mM	68°C a	RACE
TA-PAT RT template1 (for filling and tagging)	3NHC3 GCT TCA GAT CAA GGT GAC CTT TTT TTT TTT TTT TT		e	e	tagging
TA-PAT RT1 (for cDNA synthesis)	GCT TCA GAT CAA GGT GAC CTT T		e	e	cDNA synthesis
TA-PAT RT1<br>rat IMPA1-1351F	GCT TCA GAT CAA GGT GAC CTT T<br>TAG TGC AGA GCC AGT CAG ACA GTA GG		2.5mM	60ºC	PCR
Upf1-2683F<br>Upf1-2926R	TGAGCTACTACAAGGAGCAGAAGG<br>TGATCATGCTAATCTGGTCATGG	XM_003751566.4	a	a	qPCR

a: As per instruction of the polymerase mix's manufacturer

b: touchdown PCR Tann=72ºC x 5 cycles, 70ºC x 5 cycles, 68ºC x 25 cycles

c: new annotation

d: touchdown PCR Tann=72ºC x 5 cycles, 70ºC x 5 cycles, 68ºC x 20 cycles

e: not applicable

PCR and RT-PCR conditions

Initial denaturation: 94°C, 2min followed by 36 cycles [94°C, 30 sec; annealing temperature (as indicated above) 30sec; 72°C, 1min], final elongation 72°C 5min.

Quantitative RT-PCR conditions

Initial denaturation: 94°C as per manufacturer's instruction (10 min or 2 min) followed by 40 cycles [94°C, 10sec; annealing temperature (as indicated above) 20sec; 60C°C, up to 1 min; reading], melting curve T annealing to 100°C.

Antibodies and dilutions

Antibody name	Source and catalogue number	Application and working dilution
Anti-Ago2	Abcam, ab186733	WB, 1:1000<br>IF, 1:100
Anti CPSF3	Santa Cruz, sc-393001	IF, 1:100
Anti-GFP	Abcam, ab6556	IF, 1:1000<br>WB, 1:5000
Rb HA	CST, 3724	WB, 1:1000
anti-HA	CST372	IF, 1: 500
HuD	Santa Cruz, Sc-28299<br>Or<br>Santa Cruz, sc-5979	co-IP, 2μg/IP<br>RIP, 5μg/IP<br>WB, 1:2500
anti-mCherry	Abcam, ab125096	IF, 1:1000
Anti-neurofilament	Abcam, ab4680	IF, 1:3000
Anti-neurofilament	Sigma, N4142	IF, 1:500
Pabpc4	R&D	WB, 1:1000
PI3 kinase p85	Upstate, 06–497	WB: 1:2000
Hsp90	Santa cruz, sc1055	WB: 1:1000
IMPA 1	Abcam, ab184165	WB: 1:10000
anti-αtubulin	Sigma, T9026	IF: 1:1000<br>WB: 1:10000
Anti-Upf1	Millipore, 07-1014	RIP 5μg/IP<br>WB: 1:2000<br>Co-IP 2μg/IP

Applications key:Co-IP= co-immunoprecipitation, IF=immunostaining, RIP= RNA immunoprecipitation, WB= western blotting

Table S5. Related to STAR Methods. Summary statistics for RNA-seq samples alignments.

Compartment	Biological Replicates	n0. m1 [fastq]	n0. m2 [fastq]	n0. mapped	n0. alignments	n0. sequences
cell body	1	9.32E+07	9.32E+07	8.27E+07	1.68E+08	7.35E+07
	2	9.85E+07	9.85E+07	8.62E+07	1.76E+08	7.45E+07
axons	1	1.24E+08	1.21E+08	9.93E+07	1.85E+08	2.72E+07
	2	1.09E+08	1.09E+08	8.08E+07	1.55E+08	2.49E+07

m1 left end reads
m2 right end reads
